# Motor performance and functional connectivity between the posterior cingulate cortex and supplementary motor cortex in bipolar and unipolar depression

**DOI:** 10.1007/s00406-023-01671-1

**Published:** 2023-08-28

**Authors:** Lara E. Marten, Aditya Singh, Anna M. Muellen, Sören M. Noack, Vladislav Kozyrev, Renate Schweizer, Roberto Goya-Maldonado

**Affiliations:** 1 Laboratory of Systems Neuroscience and Imaging in Psychiatry (SNIP-Lab), Department of Psychiatry and Psychotherapy, University Medical Center Göttingen (UMG), Von-Siebold-Straße 5, 37075 Göttingen, Germany; 2 Cognitive Neuroscience Laboratory, German Primate Center, Kellnerweg 4, 37077 Göttingen, Germany; 3 Functional Imaging Laboratory, German Primate Center, Kellnerweg 4, 37077 Göttingen, Germany; 4 Institute of Molecular and Clinical Ophthalmology Basel, Mittlere Straße 91, 4056 Basel, Switzerland; 5 Leibniz ScienceCampus Primate Cognition, Kellnerweg 4, 37077 Göttingen, Germany

**Keywords:** Bipolar disorder, Major depressive disorder, Finger tapping, Resting state functional magnetic resonance imaging, Posterior cingulate cortex, Supplementary motor area

## Abstract

Although implicated in unsuccessful treatment, psychomotor deficits and their neurobiological underpinnings in bipolar (BD) and unipolar (UD) depression remain poorly investigated. Here, we hypothesized that motor performance deficits in depressed patients would relate to basal functional coupling of the hand primary motor cortex (M1) and the posterior cingulate cortex (PCC) with the supplementary motor area (SMA). We performed a longitudinal, naturalistic study in BD, UD and matched healthy controls comprising of two resting-state functional MRI measurements five weeks apart and accompanying assessments of motor performance using a finger tapping task (FTT). A subject-specific seed-based analysis describing functional connectivity between PCC-SMA as well as M1-SMA was conducted. The basal relationships with motor performance were investigated using linear regression models and all measures were compared across groups. Performance in FTT was impaired in BD in comparison to HC in both sessions. Behavioral performance across groups correlated significantly with resting state functional coupling of PCC–SMA, but not of M1-SMA regions. This relationship was partially reflected in a reduced PCC–SMA connectivity in BD vs HC in the second session. Exploratory evaluation of large-scale networks coupling (SMN–DMN) exhibited no correlation to motor performance. Our results shed new light on the association between the degree of disruption in the SMA–PCC anticorrelation and the level of motor impairment in BD.

**Supplementary Information:**

The online version contains supplementary material available at 10.1007/s00406-023-01671-1.

## Introduction

Psychomotor functioning involves processes that range from planning, initiating and executing movements [1, 2]. Slower or inefficient information processing may derive from alterations in particular neural regions, or entire networks, that are essential to the circuitry [3–7]. The precentral gyrus contains the primary motor cortex (M1), which is in charge of starting the execution loop of movements of the contralateral limb [8, 9]. For the coordinated adjustment of muscles implicated in the intended hand movement, the supplementary motor area (SMA) integrates signals of frontal planning with sensorial, proprioceptive and cognitive information from other brain regions and forwards it to the M1 [8–11]. Another pivotal region involved in successful motor responses is the posterior cingulate cortex (PCC), a region belonging to the default mode network (DMN) [12]. PCC plays a role in integrating signals from somatosensory areas and dorsal visual stream via parietal cortical areas for spatial processing and action in space [13]. In stroke patients with impairments in motor performance, strengthened resting state functional connectivity between the M1 and the PCC was found to be critical for improvements in motor performance [14]. This led us to hypothesize that disruptions in PCC connections might also be relevant for motor performance in other diseases that affect brain function, such as depression. Studies have shown that the PCC is also involved in emotional processing and rumination of negative thoughts [15–18], making it an relevant target of research in depression which, to our knowledge, has not been previously studied in this context.

Deficits in psychomotor functioning is a typical symptom cluster of major depressive disorder and the depressive phase of bipolar disorder [19, 20] and can make a decisive contribution to unsuccessful treatment [21, 22] as well as to social functioning [23]. In spite of their impact on patient outcomes, their manifestation is often assumed to be either part to residual symptoms or medication side effects, leaving their neural correlates and development of potentially targeted treatments poorly explored. The identification of more objective differences involving motor performance and its neural correlates in groups of bipolar depressed (BD), unipolar depressed (UD) patients and healthy controls (HC) could be a hint for developing novel biomarkers. Furthermore, such neurofunctional aberrations of bipolar disorder and major depressive disorder can offer the chance to better understand the basis of motor symptom manifestation and treatment improvement [24], for example, by applying more targeted transcranial magnetic stimulation (TMS) protocols [25].

Different paradigms can be used to assess and compare the motor performance of patients with major depressive disorder, bipolar disorder, controls and recovered patients [26–31]. In the present study, we have chosen the finger-tapping-task (FTT) paradigm, considering that it dismisses working memory [32, 33], which allows clearer conclusions about the movement process itself. It has been previously shown that motor performance in FTT is impaired in bipolar disorder [34, 35], but not always in major depressive disorder, [5, 36–40]. This reinforces an importance of understanding the underlying brain mechanisms leading to such differences. In this sense, even comparable motor impairments in major depressive disorder and bipolar disorder may be associated with differing neural correlates [41]. In light of possible residual motor symptoms [42, 43], the fundamental evaluation of motor performance, e.g. using the FTT, related to baseline M1 and PCC functional connectivity in depressed subjects undergoing treatment remains unexplored to our knowledge. Therefore, our aim with this study was to test whether resting state functional connectivity of M1 and PCC with SMA, which is accessible on the MRI scanner to all patients, regardless of how severely depressed they are, can explain behavioral differences in subjects. Functional connectivity between the right M1 and the SMA, an integrative center of the sensory motor network (SMN), can be a positive predictor of performance during the FTT in HC [44]. Thus, baseline functional dysconnectivities with the SMA could potentially explain behavioral differences between BD and UD patients in contrast to HC.

Recent studies have conceptualized the integration of the influences of affected neurotransmitters on subcortical-cortical loops as functional psychomotor brain units in major depressive disorder and bipolar disorder among other mental disorders [6, 45–52]. This concept primarily focus on explaining psychomotor impairments at the behavioral level, but the link with objective measures of psychomotor function remains to be established. Studies that unveil the functional connectivity implicated in FTT can broaden our understanding from the perspective of motor performance deficits in depression [34, 35, 39, 40]. In this regard, a connection between our regions of interest as critical part of the psychomotor system—the PCC and the SMA—is of particular interest and can contribute to the consolidation of this idea.

Thus, in the present investigation, we expect that FTT motor performance is impaired in BD and UD in relation to HC, a difference that could decrease after antidepressant treatment. The latter was addressed by longitudinal observation of treated patients. We hypothesize that the functional connectivity of M1 and PCC (our regions of interest for the seed-based analysis) with the SMA relates to FTT motor performance. Finally, we explore if the cross-correlation between the entire SMN and DMN (rather than the regions of interest) better explains differences in the level of motor performance, as a model involving the imbalance between the SMN and the DMN has been recently proposed to explain psychomotor deficits in affective disorders among others. [6, 53, 54]

## Materials and methods

### Protocol

The study was performed at the University Medical Center Göttingen (UMG) after approval of the research protocol by the local Ethics Committee. In accordance with the Declaration of Helsinki, all participants provided their verbal and written informed consent after a detailed explanation of the research protocol. Patients diagnosed by their psychiatrists with a depressive phase of major depressive disorder (UD group) or a depressive phase of bipolar disorder (BD group) (cf. ICD-10) and sex, age, and education matched HC (Table 1) were recruited by announcements at the local University, the UMG Psychiatric Center as well as the local Asklepios Psychiatric Hospital.

**Table 1:** Characteristics of the bipolar depression (BD), unipolar depression (UD), and healthy control (HC) groups

	BD	UD	HC	*p* value	Interpretation, post-hoc
*N* (% female)	21 (33.3%)	27 (40.7%)	31 (58.1%)	0.176^a^	n.s
Age (years)	43.5 ± 9.8	39.6 ± 15.3	41.7 ± 14.3	0.591^b^	n.s
Education in years	16.8 ± 4.0	15.5 ± 3.1	16.5 ± 3.8	0.453^c^	n.s
*N* lefthanders (%)	1 (4.8%)	2 (7.4%)	1 (3.2%)	–^d^	–^d^
Age of first episode	24.7 ± 9.5	29.5 ± 15.2	–	0.182^b^	n.s
Years since first episode	18.9 ± 13.5	10.1 ± 9.4	–	0.161^b^	n.s
Number of depressive episodes	7.2 ± 6.1	4.8 ± 3.8	–	0.095^c^	n.s
Number of manic episodes	2.9 ± 3.6	–	–	–	–
Number of hypomanic episodes	3.3 ± 5.0	–	–	–	–
BDI-II visit 1	21.1 ± 16.7	29.5 ± 11.1	2.6 ± 3.7	** < 0.001**^b^	Games-Howell post-hoc test: difference HC and UD: ***p* < 0.001**, HC and BD: ***p* < 0.001**, UD and BD: p = 0.128
BDI-II visit 2	17.1 ± 13.1	20.2 ± 14.0	2.3 ± 4.2	** < 0.001**^b^	Games-Howell-post-hoc-test: difference HC and UD: ***p* < 0.001**, HC and BD: ***p* < 0.001**, UD and BD: *p* = 0.714
Delta BDI-II visit 2–visit 1	− 3.9 ± 13.3	− 9.3 ± 11.9	-0.2 ± 2.6	**0.009**^b^	Games-Howell-post-hoc-test: difference HC and UD: ***p* = 0.002**, HC and BD: *p* = 0.437, UD and BD: *p* = 0.327
MADRS visit 1	13.9 ± 10.6	24.2 ± 8.6	–	**0.001**^c^	Severity of depressive symptoms is higher in UD
MADRS visit 2	10.5 ± 8.3	14.3 ± 10.2	–	0.179^c^	n.s
Delta MADRS visit 2 – visit 1	− 3.3 ± 9.3	− 9.9 ± 10.3	–	**0.028**^c^	Reduction in the severity of depressive symptoms is higher in UD
YMRS visit 1	6.1 ± 6.9	1.7 ± 2.6	–	**0.012**^b^	Severity of manic symptoms (mixed episode) is higher in BD
YMRS visit 2	2.3 ± 3.5	1.4 ± 1.7	–	0.222^b^	n.s
Delta YMRS visit 2 – visit 1	− 3.7 ± 6.2	− 0.3 ± 2.1	–	**0.025**^b^	Reduction in the severity of manic symptoms (mixed episode) is higher in BD
Medication load visit 1	4.5 ± 2.4	4.3 ± 3	0 ± 0	No *p* value because of a variance of 0 in controls^b^	Games-Howell post-hoc test: difference HC and UD: ***p* < 0.001**, HC and BD: ***p* < 0.001**, UD and BD: *p* = 0.938
Medication load visit 2	3.8 ± 2.3	4.1 ± 2.6	0 ± 0	No *p* value because of a variance of 0 in controls^b^	Games-Howell post-hoc test: difference HC and UD: ***p* < 0.001**, HC and BD: ***p* < 0.001**, UD and BD: p = 0.925
Delta medication load visit 2–visit 1	− 0.7 ± 2.5	− 0.2 ± 1.9	0 ± 0	No *p* value because of a variance of 0 in controls^b^	Games-Howell post-hoc test: difference HC and UD: *p* = 0.867, HC and BD: *p* = 0.405, UD and BD: *p* = 0.700

*p*<0.05 are shown in bold

^a^Chi-square test

^b^One-way ANOVA with Brown-Forsythe correction for inhomogeneity of variance

^c^One-way ANOVA with homogeneity of variance

^d^Analysis repeated without lefthanders

*BD* bipolar depressed, *BDI-II*  Beck depression inventory-second edition, *HC* healthy controls, *MADRS*  montgomery–Åsberg depression rating scale, *UD*  unipolar depressed, *YMRS* Young mania rating scale

All participants were between 18 and 65 years old. Exclusion criteria were contraindications for MRI scans (i.e. pregnancy, metallic implants), strong visual impairment, neurological disorder, brain surgery or head trauma, current substance abuse, past or present psychiatric disorder for HC, and comorbid psychiatric diagnosis other than anxiety disorders for BD and UD.

We collected individual information about the handedness [55] and being lefthanded was not an exclusion criterion in order to consider all the handedness variability in the clinical sample. And to consider the influence of this variable, we also complement the analyses with models excluding lefthanders.

**Fig. 1:**
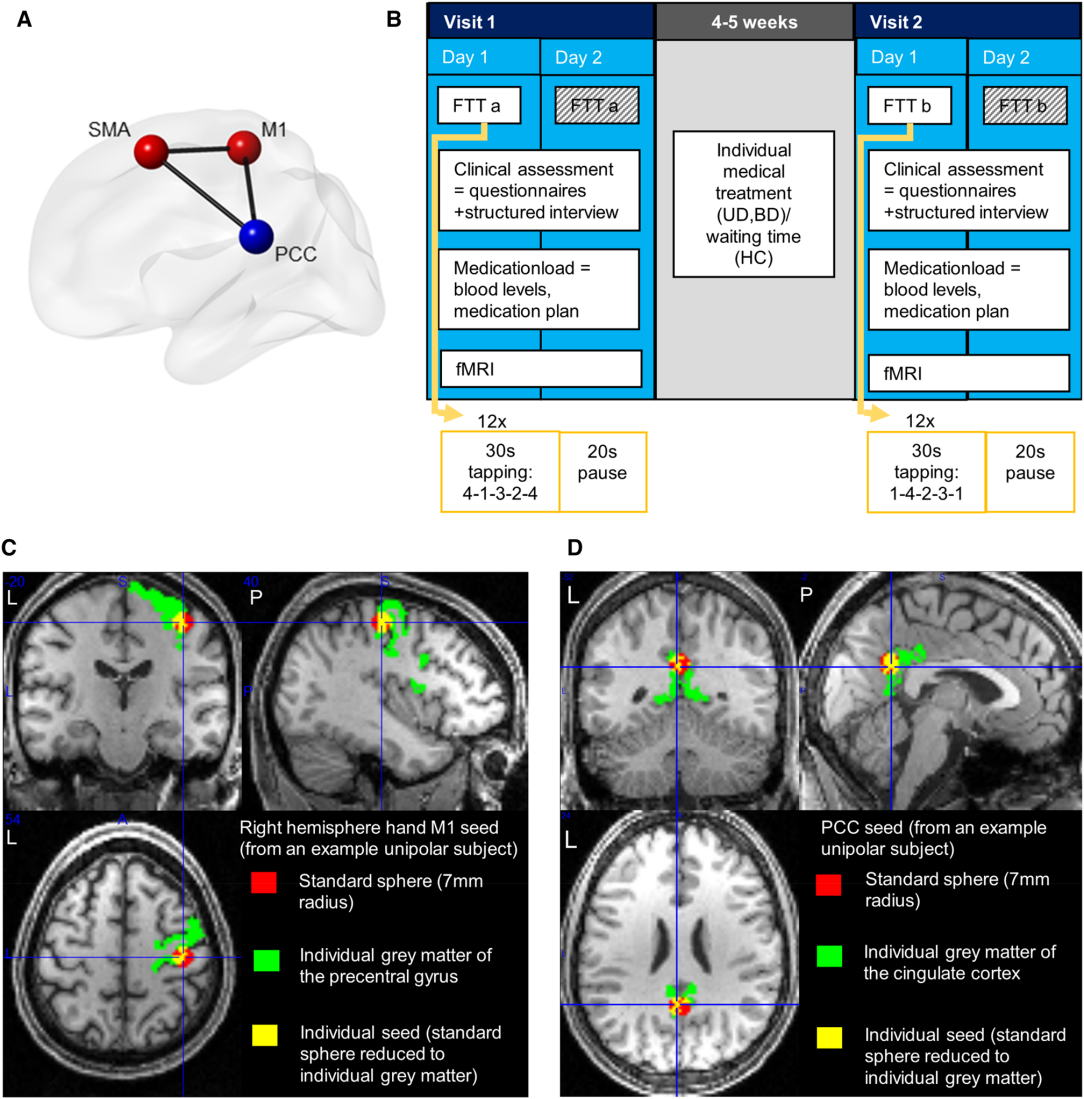
Methods: (**A**) Connectivity model between regions of interest: supplementary motor area (SMA) and right hemisphere hand primary motor cortex (M1) as part of the sensory motor network (red), posterior cingulate cortex (PCC) as part of the default mode network (blue), (**B**) Study design: unipolar (UD), bipolar (BD) depressed and healthy control subjects (HC) performed the finger-tapping-task (FTT) on both days of both visits, FTTa and FTT b versions as described in methods section. The data of the second days was not used for the analysis of motor performance. The clinical assessment and the MRI sessions took place on one of the days. The blood sample was taken as close as possible before the MRI measurement, (**C**) Seed definition technique: exemplification of the overlapping area (yellow) based on FreeSurfer individual segmentation of grey matter (green) and a spherical seed (red) at the MNI coordinates *x* = 40, *y* = − 20, *z* = 54 of functional coordinates for right hemisphere hand motor cortex (M1)^66^ (D) and at *x* = 0 *y* = − 53, *z* = 2 for the posterior cingulate cortex^14^

The study comprised two assessments of two consecutive days separated by approximately 5 weeks (Fig. 1). Between evaluations, patients received psychopharmacological treatment for the depressive episode at the psychiatric hospital by their caregiver physicians, who were independent in their treatment choices from the research group that conducted the study (naturalistic design). HC participated in the same study protocol, but received no treatment. All subjects were instructed to abstain from alcohol for at least 24 h before and to refrain from consuming nicotine or caffeine for at least two hours before the assessments. On each day the participants performed the FTT, on the second day followed by a structured interview led by a trained person and—in case of medication with measurable blood metabolites (Supplementary Table 1)—a blood sample was taken immediately prior to the MRI session to measure their blood medication levels.

Severity of symptoms in patients was assessed with the Montgomery Åsberg-Depression Rating Scale (MADRS), the Young Mania Rating Scale (YMRS), and the Beck Depression Inventory-second version (BDI-II), the first two rated within the structured interview, the latter, self-rated. We used the BDI-II in all our groups as our primary scale to implement the degree of depressive symptomatology in the analyses. The BDI-II is well correlated with other-rated scales (MADRS, Hamilton Depression Rating Scale (HAMD-17)) and has a focus on cognitive aspects of depression and less on mood and anxiety or neurovegetative aspects [56], which is in line with the research focus of this study. Furthermore, the BDI-II as a self-rated scale showed more correlation to brain metabolism in resting state positron emission tomography than HAMD as other-rated scale, indicating that BDI-II can be more strongly related to basal brain functioning [57]. To account for the amount of psychotropic medication used per patient, an extended concept previously established as medication load [58–62] was implemented (Supplementary material, item 1.1). In addition, it was ensured post-hoc that the number of subjects taking neuroleptics, which are known for their motor side effects, was comparable (supplementary material, item 2.1). In HC, to verify the absence of potential psychiatric symptoms, the self-rated Symptom Checklist-90-Revised (SCL-90-R) was used.

### Finger-tapping-task

The FTT was performed on a computer with an adapted keyboard that contained only the keys 1 to 4. Following previous research using the FTT [5, 32, 33, 36–38, 44] the subjects tapped with the digits of the non-dominant hand not using the thumb. The task consisted of 12 intervals of 30 s duration in which the tapping was performed with intervals of 20 s without digit movements between them. The entire tapping sequence was presented on the computer monitor with numbers associated with digits: 1 = little finger, 2 = ring finger, 3 = middle finger, 4 = index finger. On the two days of the first visit the sequence “4–1–3–2–4” was used, on the two days of the second visit the sequence “1–4-2–3–1” was used (Fig. 1). The participants were instructed to press the keys as fast and accurately as possible. No feedback was given on the performance of the task. The mean from the number of correctly tapped sequences of the last three of the 12 tapping intervals of the first days of the visits where used as a score to quantify the motor performance—as they are expected to represent the performance plateau [33, 63]. The number of correctly typed sequences accounts for speed and accuracy. [32, 33]

### Functional connectivity

The functional and structural images were acquired with a 3T Siemens Magnetom Tim Trio, on Syngo VB-17a (Erlangen, Germany) and a 32-channel head coil. Structural whole-brain T1-weighted images were realized with a magnetization prepared rapid gradient echo (MPRAGE) sequence (repetition time: 2250 ms, echo time: 3.26 ms, inversion time: 900 ms, flip angle: 9°, field of view: 256 mm, spatial resolution: 1 mm × 1 mm, 176 slices, acquisition time: 8:26 min) and a resulting voxel size of 1 mm × 1 mm × 1 mm.

Resting state functional MRI (rs-fMRI) was measured with gradient echo EPI (echo planar imaging) sequence (repetition time: 2500 ms, echo time: 33 ms, flip angle 70°, multi-band-factor 3, field of view 210 mm, spatial resolution: 2 mm × 2 mm, 60 slides with 2 mm thickness, distance factor 10%, 125 volumes (first 5 volumes deleted)) with a total acquisition time of 5:25 min, voxel size of 2 mm × 2 mm × 2 mm and a slice orientation along the anterior–posterior commissure. When measuring the rs-fMRI, participants were asked to keep their eyes open, fix their gaze on a fixation cross displayed on the screen, not sleep, and let their mind wander.

Brain images were analyzed with SPM12 (www.fil.ion.ucl.ac.uk/spm, Wellcome Trust Centre for Neuroimaging, London, United Kingdom) based on MATLAB 2015b (MathWorks, Natick, Massachusetts) The following preprocessing steps were performed: default temporal high-pass filtering SPM12, slice-time-correction, realignment with unwarping SPM12; spatial normalization with the deformation field from FreeSurfer Version 6.0 (https://surfer.nmr.mgh.harvard.edu/) run on an Ubuntu 18.0 to the standard Montreal Neurological Institute (MNI) 152 brain at 2 mm spatial resolution; spatial smoothing with a Gaussian kernel of 6 mm full width at half maximum (FWHM); nuisance-regression of white matter and cerebral spinal fluid as well as motion correction with an automatized independent component analysis based cleaning procedure (ICA-AROMA v0.3-bet, available from: https://github.com/maartenmennes/ICA-AROMA) [64]. To ensure all autocorrelative signal was regressed out, a visual inspection of all data for quality control was performed. If potential noise artefacts remained, they were manually added as additional “noise” components to the automatically identified by ICA-AROMA and the cleaning procedure was rerun. In addition to this extensive correction of movement we also assessed potential bias due to different extend of movement between the groups by comparing individual root mean squares based on frame-by-frame displacement of adjacent volumes in mm between the groups in an repeated measures ANOVA (rmANOVA). [65]

Seed-based analyses aimed to address functional connectivity differences and its influences on motor performance of the right hemisphere hand M1 and the PCC, especially to the SMA as the core region of our hypotheses. To define the M1 gray matter seeds, we combined the anatomical T1-weighted information from the precentral gyrus from FreeSurfer segmentation with a 7 mm radius spherical seed, defined using the SPM toolbox MarsBaR version 0.44 (http://marsbar.sourceforge.net/), which was centered at the MNI-coordinates 40–20 54 (*x*, *y*, *z*) for the right hemisphere hand M1 from a meta-analysis of functional MRI data of hand movement tasks [66]. The overlap between these two sources was carefully inspected for consistency in every individual subject’s data (see an example for precise coverage of the grey matter with a seed-based on this technique in Fig. 1). To make sure that differences in functional connectivity did not stem from differences in the seed volume, we checked if the volume of the individualized masks differed between groups or time with an rmANOVA in SPSS (for results see Supplementary material, item 2.2). The subject-specific seed technique, as described for M1 seeds, was also applied to create seeds for the PCC (Fig. 1) from an overlap of a sphere centered in the position of a previous study investigating motor impairments in stroke (MNI coordinates 0 − 53 26 (*x*, *y*, *z*)) [14] and the individual FreeSurfer cortical segmentation.

The time courses of these seeds were bandpass filtered (0.01–0.1 Hz) before being included into a general linear model (GLM) in SPM12 for the first level analysis. According to the known task-positive and task-negative activations in the right hemisphere hand M1 and PCC respectively, we focused on brain regions positively correlated when seeding in the right hemisphere hand M1 and regions negatively correlated (anticorrelated) when seeding in the PCC.

To investigate potential full network interconnection deficiency between SMN and DMN in depressive patients, a group independent component analysis (gICA) was performed using MELODIC Version 3.14 in FSL (FMRIB’s Software Library, www.fmrib.ox.ac.uk/fsl) [67], to be able to estimate correlation between the time courses of these networks in all groups before and after intervention. The gICAs were performed for each group and each visit separately. Then, all the components were visually inspected to select the components representing SMN, anterior DMN and posterior DMN. The time course of the network signal from every subject was extracted separately for each selected component. For every subject a correlation coefficient for the correlation between the time courses of the SMN and the anterior DMN as well as for the correlation between the SMN and the posterior DMN was calculated using MATLAB 2015b (MathWorks, Natick, Massachusetts).

### Statistical analysis

The FTT was analyzed with an rmANOVA for motor performance in IBM SPSS Statistics Version 26. In the main analysis no covariates were included, but to check for influencing effects, the analysis was repeated with the covariates sex, age, education, change of the medication load and change in BDI-II, which have the potential to influence the motor performance [22, 68–72]. Furthermore, models with only the patient groups were created to consider potential effects of years since first episode, age of onset, number of depressive episodes, change in MADRS and change in YMRS, which have been described in the literature [26, 70]. The statistical threshold for the tests was set to two-tailed *p* < 0.05. If the assumption of homogeneity of variance was met for the ANOVAs, we used the Bonferroni post-hoc-test to correct for multiple testing. But if this assumption was not met, we used the Games-Howell post-hoc-test.

For the rs-fMRI data, a linear regression of seed-based functional connectivity and motor performance was calculated in SPM for the entire study sample at both visits. Additionally, accounting for dependence on longitudinal measures, two full factorial models were built in SPM with group and time as factors. The two models were created with and without medication load as a covariate-of-no-interest to control for possible differences arising from the medication load that the patients received. Additionally, a linear regression was performed for the entire study population at both visits in SPSS to test for a relationship between the SMN-DMN time course correlations and the motor performance. The SMN-DMN correlation coefficients were used for an rmANOVA model calculated in SPSS to investigate whether possible correlations between the networks differed between groups or time.

In regions belonging to our a priori hypothesis, we considered the statistical threshold at the cluster-wise correction level (*p* FWEc < 0.05) to reduce the probability of both false-positive and false-negative results. Otherwise, we considered the family-wise error correction level (*p* FWE < 0.05) to reduce the probability of false-positive results. The labeling of functional imaging clusters was performed according to the automated anatomical labeling (AAL) atlas. [73]

## Results

From 95 participants, 11 were unable or unwilling to complete the entire study, one participant performed the FTT with the wrong hand and four participants were excluded due to abnormalities in the anatomical scans, e.g. intracranial epidermoid cysts. Therefore, data from 79 participants (31 HC, 27 UD, 21 BD–18 Type I, three Type II) were taken into final analysis. One participant from each of the HC and BD groups and two participants of the UD group were lefthanded.

### Characterization of groups

The mean time between the two visits was 4.9 weeks (SD 0.8). A complete evaluation of the characteristics between groups is presented in Table 1. In line with our matching, the groups were comparable in terms of sex, age and years of education. The patient groups did not differ in terms of the history of their disease measured by age of onset, years since first episode and number of depressive episodes. The HC did not exceed the threshold of normality in the global severity index of the SCL-90-R score.

The severity of the depressive episode was evaluated during the study and at both visits the patient groups showed comparable depressive episode severity according to the BDI-II scores (Table 1). An rmANOVA with the BDI-II scores revealed a significant change between sessions and patient groups (factor “time F(1,76) = 15.660, *p* < 0.001; interaction “group x time” F(2,76) = 6.035, *p* = 0.004; factor “group” F(2,76) = 41.138, *p* < 0.001). The comparison of the difference in BDI-II scores of visit 1 and 2 between groups revealed a significantly score reduction for UD compared to HC.

In MADRS, the severity of depressive symptoms was significantly higher in UD, but only at the first visit (Table 1). An rmANOVA with the MADRS scores revealed a significant reduction between visit 1 and 2 for both patient groups but stronger in UD than in BD (factor “time” F(1,46) = 21.067, *p* < 0.001; interaction “group x time” F(1,46) = 5.179, *p* = 0.028; factor “group” F(1,46) = 8.903, *p* = 0.005). There was a significant difference seen for YMRS scores with BD showing higher scores and a stronger reduction across time than UD in an rmANOVA (factor “time F(1,46) = 10.067, *p* = 0.003; interaction “group x time” F(1,46) = 7.024, *p* = 0.011; factor “group” F(1,46) = 7.491, *p* = 0.009).

**Fig. 2:**
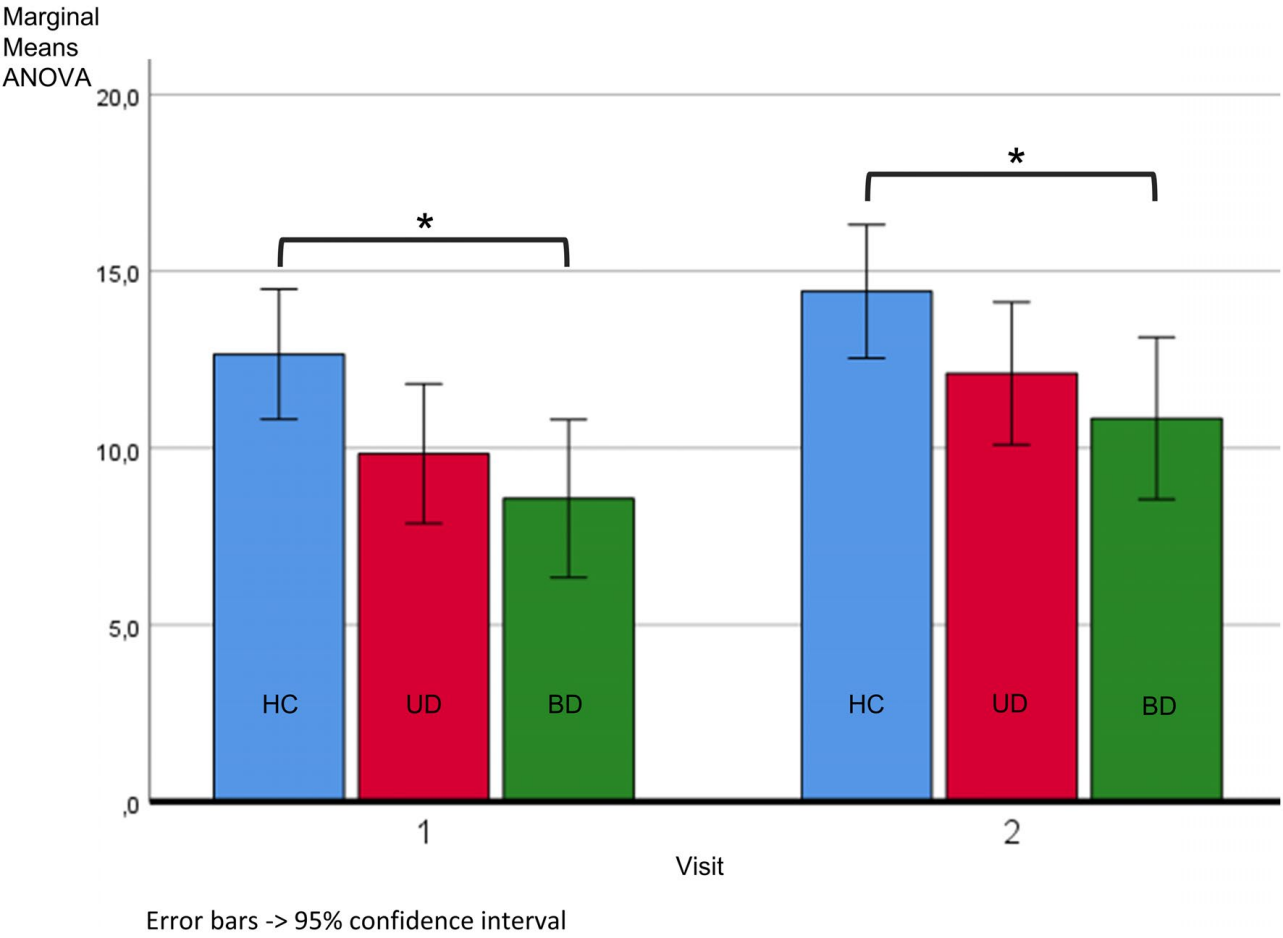
Motor performance scores: Marginal means (mean number of correctly tapped sequences per 30 seconds) and confidence intervals from repeated-measures ANOVA of motor performance scores for healthy controls (HC, blue), unipolar (UD, pink) and bipolar (BD, green) patients. * = *p* < 0,05; The values displayed in the graph are HC visit 1 M ± SD = 12.7 ± 6.1, UD visit 1 M ± SD = 9.8 ± 4.2, BD visit 1 M ± SD = 8.6 ± 4.6, HC visit 2 M ± SD = 14.4 ± 5.8, UD visit 2 M ± SD = 12.1 ± 5.0, BD visit 2 M ± SD = 10.8 ± 4.7. Significant differences are seen only between HC and BD (ANOVA factor “group” F(2,76) = 4.122; *p* = 0.020) with BD showing a significantly reduced number of correctly performed finger tapping trials compared to the HC (Post-hoc-test *p* = 0.023)

Although only bipolar patients in the current depressive episode were recruited, the symptoms were mixed in some patients. At the first visit, three bipolar patients showed subclinical depressive symptoms (BDI-II score ≤ 8 and MADRS score ≤ 12), five showed manic symptoms (YMRS score > 9) and one showed both manic (YMRS score of this subject at visit 1 = 14) and depressive (BDI-II score of this subject at visit 1 = 53; MADRS score of this subject at visit 1 = 28) symptoms. Even though being subclinical (*M*_YMRS_ ≤ 9), the BD group showed a significantly higher YMRS score on visit 1 compared to the UD, as well as a greater change in YMRS score between the two visits. To confirm that the main results were not driven by the individuals with manic symptoms characteristics or subclinical depressive symptoms characteristics, we compared mean motor performance scores of the original BD group, with a subgroup of BD with clinical depressive symptoms only, which showed comparable values (BD visit 1 = 8.6 ± 4.6 (M ± SD) (Fig. 2); BD visit 1 with clinical depressive symptoms only: 8.4 ± 4.9 (M ± SD)). We also repeated the imaging analysis of linear regression of motor performance scores and functional connectivity of PCC seeds without the BD subjects showing manic, mixed or subclinical symptoms and the main results could still be identified.

No difference in medication load between patient groups was seen at visit 1 or visit 2, but the load in both visits was significantly different from zero, represented by the not medicated HC group. Furthermore, the change in medication load between visit 1 and 2 was small in the patient groups (Table 1) and not distinguishable from zero, represented by the HC group on an additional rmANOVA (factor “time” F(1,76) = 2.431, *p* = 0.123; interaction “group x time” F(2,76) = 1.155, *p* = 0.320; factor “group” F(2,76) = 48.226, *p* < 0.001). For a detailed description of medication taken by the patient groups see item 2.1 in the Supplementary material.

### Behavioral results

The motor performance differed between groups (ANOVA factor “group” F(2,76) = 4.122; *p* = 0.020; Fig. 2) with BD showing a significantly reduced number of correctly performed finger tapping trials compared to the HC (Post-hoc-test *p* = 0.023). The UD group showed no performance impairment compared to HC (Post-hoc-test *p* = 0.158). The BD and UD showed no significant difference in motor performance (Post-hoc-test *p* = 1).

All groups showed an improvement of their motor performance in visit 2 (ANOVA factor “time” F(1,76) = 33.800; *p* < 0.001). The improvement did not differ between the groups (interaction “group x time” F(2,76) = 0.230; *p* = 0.795).

By including the covariates sex, age, education, change of medication load and change in BDI-II into the model, the difference in performance between the groups remains significantly different (factor “group” F(2,71) = 5.194; *p* = 0.008) as does the comparable performance improvement of the groups across the two visits (interaction “group x time” F(2,71) = 0.067; p = 0.935). However, the general effect of the performance improvement seen across the groups in visit 2 was not present anymore (factor “time” F(1,71) = 0.002; *p* = 0.966).

Influences of covariates on the motor performance in general revealed a negative correlation with age (F(1,71) = 23.542; *p* < 0.001) and a positive correlation with education (F(1,71) = 16.183; *p* < 0.001) for all groups. When limiting the ANOVA to the patient groups, motor performance did not differ between the groups (factor “group”; F(1,46) = 1.010; *p* = 0.320), but an improvement across visits was seen (factor “time” F(1,46) = 23.042; *p* < 0.001). However, it showed no interaction with groups (interaction “group” x “time”, F(1,46) < 0.001; *p* = 0.999). These results were maintained, when including the covariates years since first episode, age of onset, number of depressive episodes, change in MADRS and change in YMRS. An older age of first episode (F(1,41) = 5.808; *p* = 0.021) and more years since first episode (F(1,41) = 4.396; *p* = 0.042) reduced the motor performance. An increase in YMRS score was positively correlated with a change in motor performance (interaction “time x deltaYMRS” F(1,41) = 4.525; *p* = 0.039), a change in MADRS was not (interaction “time x deltaMADRS” F(1,41) = 0.799; *p* = 0.377). The number of depressive episodes showed a negative correlation with change in performance (interaction “time x number of depressive episodes” F(1,41) = 8.489; *p* = 0.006).

All results hold, when excluding the lefthanders from the analysis.

### Functional connectivity

#### PCC seed-based functional connectivity

**Fig. 3:**
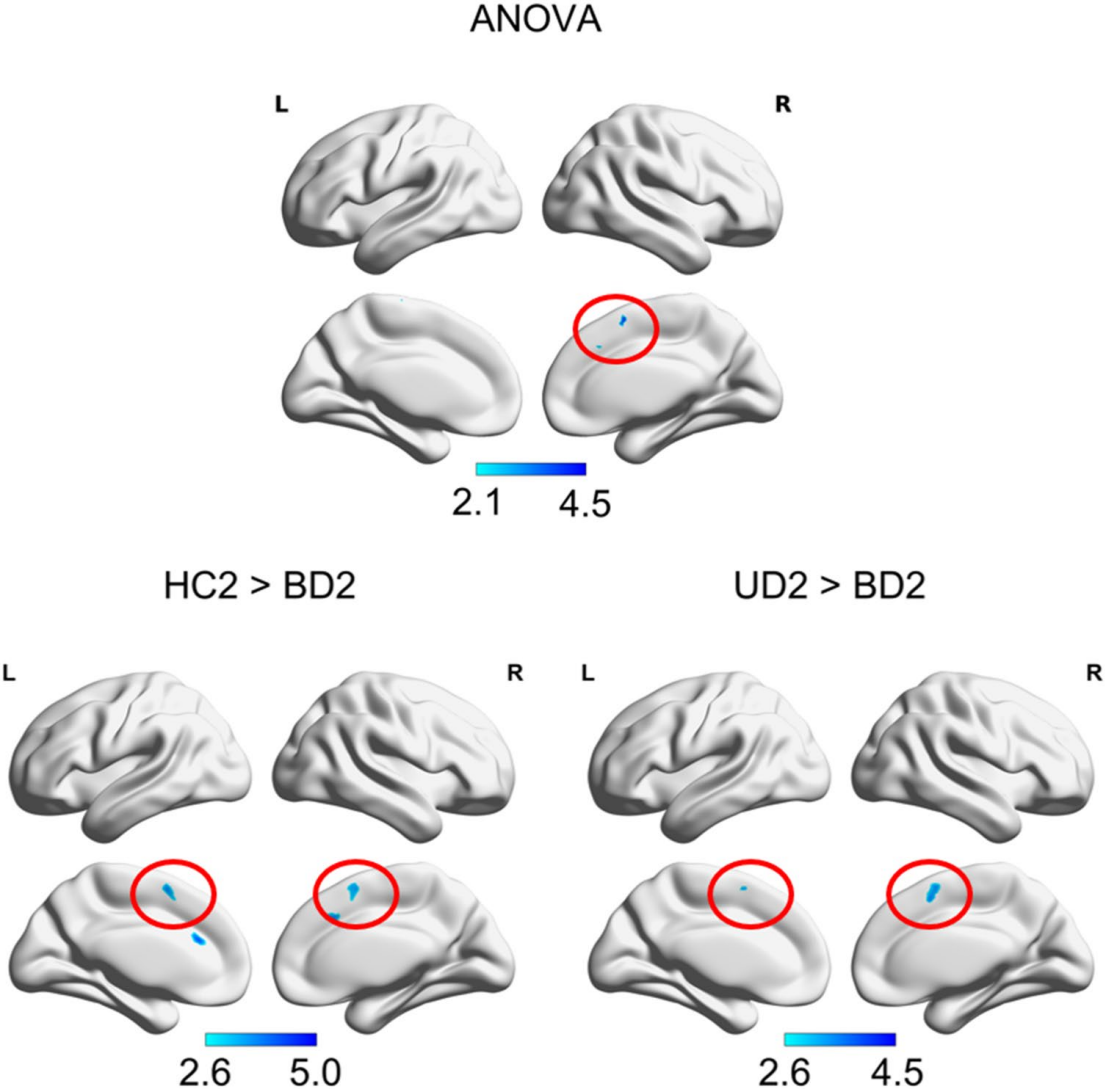
PCC connectivity differences: Resting state functional connectivity differences of the posterior cingulate cortex (PCC) ANOVA “main effect” contrast and pairwise differences in healthy controls, bipolar and unipolar depressed at the second visit (HC2, BD2, UD2, *p* FWEc < 0.05) as seem in an SPM12 full factorial model. HC2 showed higher connectivity measured by anticorrelation between the PCC and the bilateral SMA (red circle) than BD2. UD2 showed higher connectivity measured by anticorrelation between the PCC and the bilateral SMA (red circle) than BD2. Colorbar represents t-values

For each group, the seed-based functional connectivity between the PCC and the SMA was visible as a negative correlation at *p* FWE < 0.05 level, except for the BD group at visit 2. For BD, the connectivity between PCC and SMA was lower in contrast to HC, as well as to UD, at *p* FWEc < 0.05 level (Fig. 3). No group differences in the functional PCC-SMA connectivity were observed at visit 1.

**Fig. 4:**
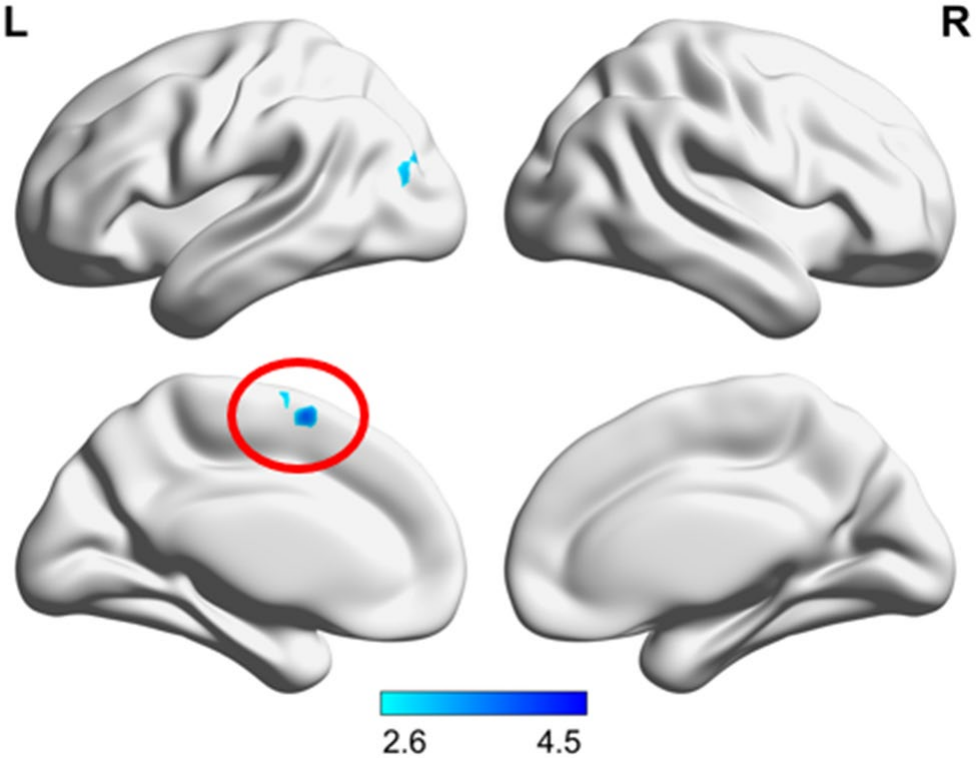
PCC connectivity correlated with motor performance Resting state functional connectivity of the posterior cingulate cortex (PCC) linearly correlated with the motor performance in the finger-tapping-task (*p* FWEc < 0.05). The motor performance of all participants is correlated with the connectivity (anticorrelation) strength between the PCC and the left supplementary motor area (SMA, red circle) as seen in an SPM12 linear regression model. Colorbar represents t-values

The linear regression of the PCC-SMA functional connectivity and the motor performance scores from both visits and all groups together revealed a positive correlation (Fig. 4). Due to the focus on regions anticorrelated with the PCC in the first level analysis, this means that the higher the performance the stronger the anticorrelated functional connectivity between PCC-SMA. When excluding the lefthanders from analysis the cluster in the SMA maintained at a level of *p* < 0.001 uncorrected, but did not survive FWEc < 0.05 level anymore, most likely due to the reduced power by the lower number of subjects in the analysis.

This is in contrast to the seed-based connectivity between PCC and M1, were no negative correlation was identified at *p *FWE < *0.05* level in all groups and visits. Also, the linear regression of the PCC-M1 functional connectivity and the motor performance scores showed no linear relationship.

**Fig. 5:**
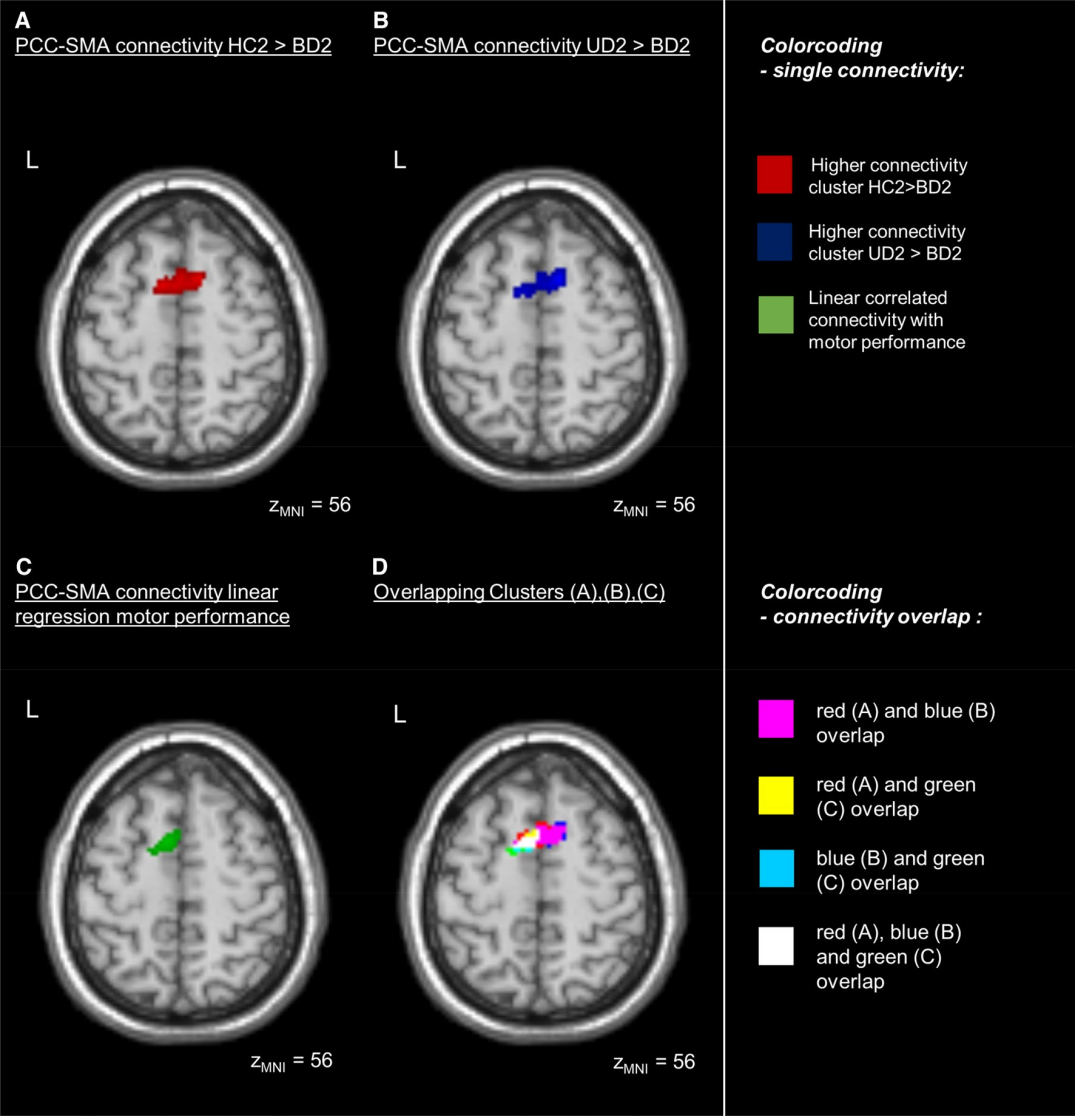
PCC connectivity overlaps in the SMA: Resting state functional connectivity differences of the posterior cingulate cortex (PCC) between groups and connectivity of the PCC correlated with motor performance overlap in the supplementary motor area (SMA) at an axial plane (z_MNI_ = 56, *p* FWEc < 0.05). **A** Healthy control subjects (HC2) show higher connectivity than the bipolar depressive subjects (BD2) at visit 2. **B** Unipolar depressive subjects (UD2) present higher connectivity than the bipolar depressive subjects (BD2) at visit 2. **C** The connectivity of the PCC to a part of the left SMA is correlated with the motor performance in the finger-tapping-task. **D** Connectivity clusters seen in (**A**, red), (**B**, blue) and (**C**, green) overlap in different extends of the SMA, as depicted in pink (A⋂B), located at medially in both hemispheres, in yellow (A⋂C), located anterior of the white area in the left hemisphere, in turquoise (B⋂C), located posterior to the white area in the left hemisphere, and in white (A⋂B⋂C), located in the left hemisphere between pink, yellow, and turquoise

Taken together and supporting the idea that lower PCC-SMA anticorrelation in BD is related to their behavioral impairment in the second session, a partial overlap in SMA is seen between the functional connectivity differences with other groups and the linear regression with motor performance (Fig. 5). This indicates that part of the SMA in BD relates to both, the reduced performance in FTT and reduced functional connectivity to PCC, but not to M1.

Positive correlation to the PCC was evidenced for regions of the DMN, namely angular gyri, precuneus, cingulate gyri, and middle prefrontal regions (data not shown), confirming the PCC as a brain region associated with the DMN.

Lastly, all findings from the PCC seed-based analysis are sustained, when medication load is included in the full factorial models as a regressor-of-no-interest.

#### M1 seed-based functional connectivity

The positive functional connectivity between the right hemisphere hand M1 and the SMA could be shown at *p* FWE < 0.05 for each of the groups. Additionally, other parts of the SMN, namely the pre- and postcentral gyrus bilaterally as well as bilateral temporal regions were identified. No negative correlation was evidenced at *p* FWE < 0.05 (data not shown).

The linear regression of motor performance score and the right hemisphere hand M1-SMA functional connectivity from both visits and all groups together did not show SMA clusters that survived *p* FWEc < 0.05 correction, i.e. there was no correlation between the motor performance and functional connectivity between SMA and M1. This result remained when excluding the lefthanders from the model.

A higher connectivity between the M1 and the SMA was seen in HC compared to UD only at visit 2 (*p* FWEc < 0.05) in the model where the medication load was inserted as regressor-of-no-interest. When excluding the lefthanders from the model with medication load as a regressor-of-no-interest, a higher connectivity between the right hemisphere hand M1 and the SMA was also seen at visit 1 at *p* FWEc < 0.05 threshold.

**Fig. 6:**
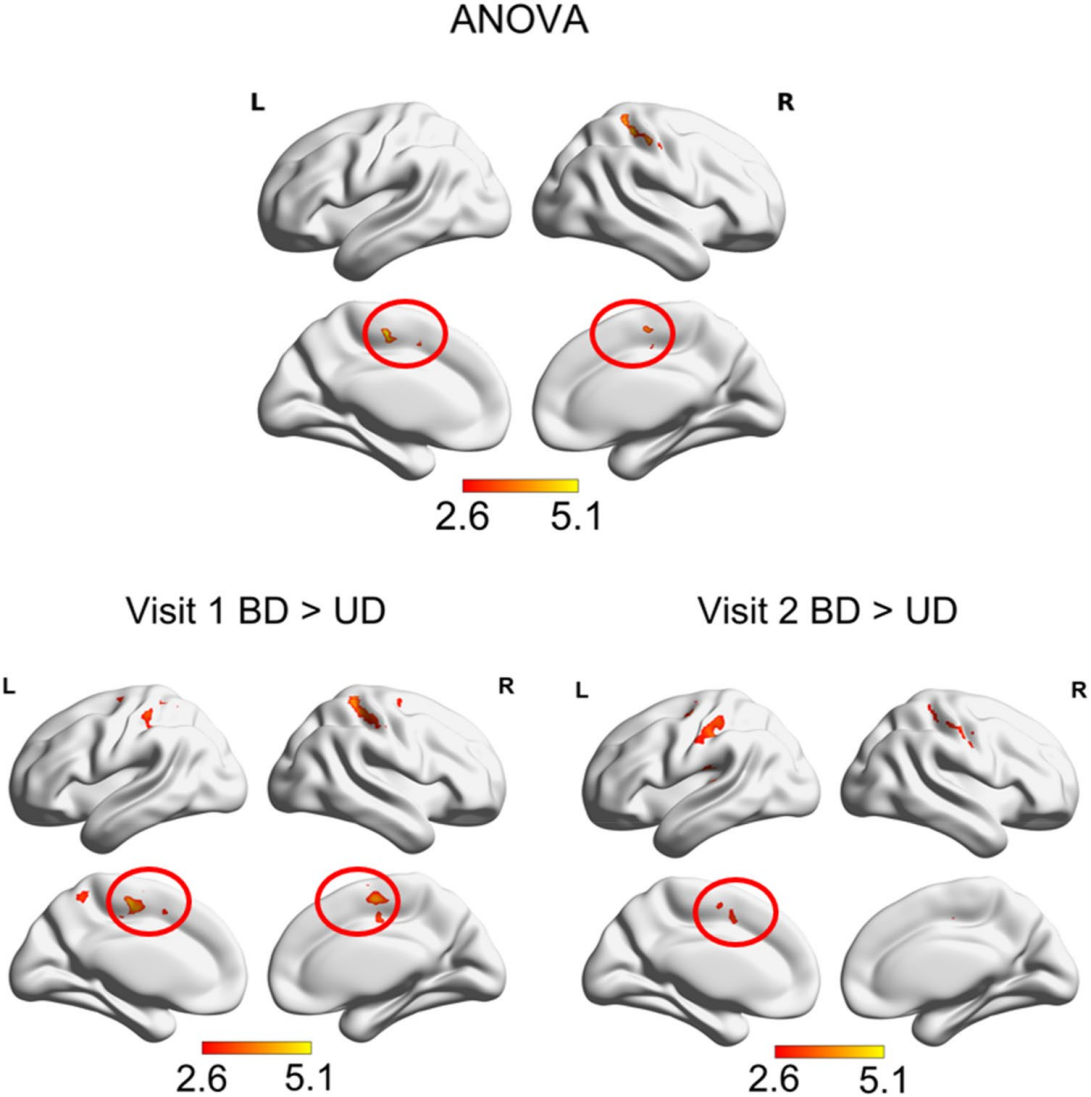
M1 connectivity differences: Resting state functional connectivity differences of the right hemisphere hand motor area (M1) ANOVA “main effect” contrast and pairwise differences between bipolar and unipolar depressed at first and second visits (BD, UD, *p* FWEc < 0.05) as seem in an SPM12 full factorial model. BD showed stronger connectivity than UD between M1 and the bilateral SMA (red circle) in visit 1 than in visit 2. No regions were evidenced in the UD>BD contrast. Colorbar represents t-values

Compared to UD, BD showed stronger connectivity between M1 and SMA on both visits (Fig. 6). At the first visit, these clusters survived *p* FWE < 0.05 correction. At the second visit, differences were more restricted and seen only in the left hemisphere at *p* FWEc < 0.05 level. These differences between BD and UD held in the model with medication load as a regressor-of-no-interest as well as in the model without lefthanders. There was no connectivity of the M1 that was stronger in UD than in BD.

Changes in right hemisphere hand M1-SMA connectivity over time were not evidenced in any of the 3 groups. Beyond our a priori hypothesis, higher connectivity of M1 with the left temporal middle gyrus was seen at visit 2 compared to visit 1 in BD (*p* FWE < 0.05) in both models with and without medication load. However, the cluster was not sustained when excluding lefthanders from analysis. Other functional connectivity changes across time were not observed. Also, beyond our a priori hypotheses, higher connectivity was seen in HC than UD at visit 2 between the right hemisphere hand M1 and the right Rolandic operculum (*p* FWE < 0.05). When controlling for the medication load as a regressor-of-no-interest, the cluster at the right Rolandic operculum was not seen. Furthermore, the cluster was not present, when excluding the lefthanders from the analysis. There was no further difference between BD and HC in any of these models.

Finally, to check for potential bias in the seed model, we evaluated whether differences in movement exists during the scanning sessions, in spite of applying a strict motion correction procedure in the analysis. When comparing the frame-by-frame displacement with root-mean-squares [65] between the three groups, a difference was revealed, which do not seem to stem from a difference between the HC-BD groups (post-hoc *p* = 0.257), but rather from a trend between the UD-BD groups (post-hoc *p* = 0.062) (for detailed results see Supplementary material, item 2.3).

### SMN-DMN correlation coefficients

The correlation coefficients between SMN and DMN were computed and compared between groups and across time in rmANOVAs (for results see Supplementary material, item 2.4, Table 6). In the linear regressions, the motor performance scores neither showed a relationship with the correlation of SMN and posterior DMN (*r* = 0.002, *p* = 0.571), nor with the correlation of SMN and anterior DMN (*r* = 0.003, *p* = 0.361). Similar to the models with lefthanders, no linear relationship between SMN-DMN correlation and motor performance was seen.

## Discussion

The current study examined the neural underpinnings of motor performance between groups of BD, UD and HC participants with comparable sex, age, education, medication load and history of disease. We identified a deficit in the FTT motor performance only in BD in relation to HC, which decreased but remained significant after five weeks of psychopharmacotherapy. As expected, an association between the FTT motor performance and the negative functional connectivity between PCC and SMA was evidenced, which in the explorative investigation did not extend to the relationship between DMN–SMN correlations. Contrary to our expectations, an association between FTT motor performance and positive functional connectivity between M1 and SMA was not observed, suggesting that the contribution of M1 is less relevant than that of PCC in this case.

Although the motor circuit is one of the best described in the literature [74], functional brain alterations in motor areas as part of depressive episodes remain poorly explored. To our knowledge, this is the first time that PCC–SMA connectivity has been evaluated and identified as critical for motor performance in a study involving depressive and healthy subjects. In line with a former study showing the importance of the PCC–SMA connectivity for upper limb motor tasks [14], we were able to show here that this connectivity also appears critical for the FTT performance. Since functional connectivity between PCC–SMA is linearly correlated with the FTT performance, which is impaired in BD compared to HC, one can assume that the functional brain difference between groups would explain the behavioral difference in the FTT. Despite these findings are promising for the identification of a brain-behavioral relationship, it must considered that such results do not survive multiple testing correction (FWEc < 0.05), when excluding the lefthanders. However, they remain identifiable at the threshold *p* < 0.001, which supports the notion of a drop in the power to detect statistical differences as cases decrease rather than left handed bias.

Psychomotor alterations are commonly seen in bipolar disorder and sometimes in major depressive disorder compared to controls, which has been suggested as a factor that negatively contributes to treatment response or even remission [21, 22]. Therefore, this remains a very relevant topic of research. In line with a former study comparing psychomotor performance in major depressive disorder, bipolar disorder and controls [30], we report evidence that patients with bipolar disorder are more likely to have an impairment in motor performance measured by the FTT. This finding is also in line with the literature showing more robust FTT impairment in patients with bipolar disorder [34, 35] than in patients with major depressive disorder, where it appears compromised only in some study samples [5, 36–40]. Overall, learning effects seem to be present from the first FTT assessment to the second, differently from previously shown [75]. In a keyboard naive population from Kenya, it was seen that such learning effects only appeared in healthy controls and not in depressive subjects [38]. A possible explanation for the improvement in the FTT performance could be that depressive patients in our study were able to profit from treatment, which may have reflected at least partially in their performance. This is an interesting hypothesis involving possible neuroplasticity changes that could be addressed in the future.

The hypothesis that the resting state functional connectivity between the M1 and the SMA is not only critical for the motor performance in HC [44], but also in UD and BD, was rejected since the linear regressions with motor performance did not show correlated clusters in the SMA when seeding the right hemisphere hand M1. As far as we know, this work is the first providing evidence that right hemisphere hand M1-SMA connectivity is not critical for influencing FTT motor performance in depressive subjects. Another possibility for negative findings is that the seed method in Herszage et al*.* [44] when detecting the functional representation of the hand with transcranial magnetic stimulation for each subject, could have been more precise. Since our method of seed determination includes not only functional but also individual anatomical information, it would be interesting to compare the two seeding methods in future studies to define the most promising way of detecting individual seeds.

Our results show that BD have a higher M1-SMA connectivity than UD at both timepoints. Since we did not find behavioral differences in the FTT between the two patient groups, we can only hypothesize that the M1-SMA connectivity might have relevance for other symptoms of depression that were beyond the scope of this work. Groups, both BD and UD were comparably depressed, in a moderate level according to BDI-II scores, but only the UD improved significantly in the second evaluation, which might be a limitation when comparing the longitudinal changes during treatment. We speculate that residual psychomotor symptoms in BD, including the differences seen in neural correlates, may contribute for the limited improvement since psychomotor retardation is known as a negative predictor for antidepressant treatment response. [76]

All individual seeds used for M1 and PCC functional connectivity analysis have been carefully created and inspected for structural accuracy of each individual anatomy aiming at the participant’s hand knob. Furthermore, their volumes did not differ across groups, which per se could lead to false conclusions. Finally, we carefully controlled our main findings for medication load and movement during scanning sessions, where we found no evidence that our main findings might have been biased from these covariates. A trend difference of movement between groups should be kept in mind. To best remove potential bias stemming from movement, we used movement correction with ICA–AROMA which was shown to be very efficient [77] and extended it with manual checking for artificial signals, so we believe that our results from BD-UD comparison are not biased by movement.

In our view, our results also open new research perspectives for the comparisons between patient groups. For example, it may be interesting in the future to use motor paradigms that comprise a motor planning component, which may be more sensitive in detecting differences in behavior between UD and BD. This approach can be useful in new ways to support early differential diagnosis where there is a clear clinical need [78]. Considering the PCC resting state connectivity in major depressive disorder, aberrations were shown as an early marker of depression [79].

It was postulated that the imbalance between the SMN and the DMN is critical for motor performance in depression [6, 53, 54]. Therefore, it might be expected that the interconnection between the SMN and DMN, as a task positive and a task negative network respectively [80, 81], is critical for motor performance in the FTT. To explore whether this model could explain the motor performance differences in a more consistent way than our seed model would, we computed individual correlation coefficients between SMN and DMN. The idea that the SMN–DMN connectivity is critical for FTT was rejected, since no linear relationship between correlation coefficients and the motor performance were revealed. It is possible that a model taking into account the imbalance between whole networks containing many regions is likely to oversimplify more complex neural mechanisms in this case.

Another postulated model for psychomotor processes consists of three units [50]. First, the “external unit”, which receives exteroceptive influences from the environment and processes them into somatomotor outputs. Second, the “internal unit” for interoceptive input and visceromotor output. Third, the “associative unit”, which incorporates thought processes associatively into the processing. M1 and SMA could be assigned to the “external unit” and the PCC to the “associative unit” respectively. [12, 50, 82, 83] If so, the interconnection between these units could be seen as critical for motor performance and perhaps useful for the differentiation of UD and BD. In the recent literature, the SMN, to which SMA belongs, has been shown to be affected by subcortical-cortical connectivities with the thalamus, raphe nucleus and substantia Nigra in different phases of bipolar disorder [46]. Even though out of the scope of our study, this realm may be investigated in future long-term longitudinal studies applying FFT in the same group of bipolar disorder to provide further insights into the phases of their disease. For example, this may help to elucidate whether PCC-SMA connectivity is a general or phase-specific characteristic of bipolar disorder, advancing our understanding of the neural correlates of psychomotor abnormalities in mania and depression.

Some limitations need to be mentioned so that our results can be viewed with caution in certain circumstances. Our sample size is comparable to other studies, but likely modest taking into account the limited power in detecting differences when excluding the lefthanders. Therefore, a replication of our findings with larger samples is prudent [84]. Another important aspect is the use of medication, which has been very tightly controlled, but still could have influenced the results in unpredictable ways. Probably due to fast switches in mood, not all BD were rated with depressive symptoms at a clinical threshold at the day of testing and some also showed a combination of depressive and manic symptoms (mixed episode). Thus, some heterogeneity can be seen as a limitation of this study, but, on the other hand, reflects clinical reality. Noteworthy, considering that BD can be often misdiagnosed as UD [78], we observed the occurrence of manic symptoms in one patient part of the UD group, for whom the YMRS was in the clinical range. Nevertheless, all patients included in this study had a long history of disease and a diagnosis confirmed by multiple caregivers of the clinical setting, which reduces the risk of a BD being misdiagnosed as UD [78, 85].

In conclusion, our seed model supports the notion that functional connectivity between PCC-SMA explains the FTT performance in healthy and depressed individuals, but the functional connectivity between M1-SMA does not. Reinforcing the relevance and originality of these findings, the SMN-DMN correlation did not explain motor performance as the seed model.

### Supplementary Information

Below is the link to the electronic supplementary material.

Supplementary file1 (DOCX 109 KB)

## Data Availability

The data supporting the findings of this study are available from the corresponding author upon request. Due to restrictions in the data sharing consent obtained from the study participants the data are not publicly available.

## References

[1] Gardini S, Venneri A, McGeown WJ (2016). Brain activation patterns characterizing different phases of motor action: execution. Choice Ideat Brain Topogr.

[2] Grafton ST, de Hamilton AFC (2007). Evidence for a distributed hierarchy of action representation in the brain. Hum Mov Sci.

[3] Witt ST, Laird AR, Meyerand ME (2008). Functional neuroimaging correlates of finger-tapping task variations: an ALE meta-analysis. Neuroimage.

[4] Liberg B, Adler M, Jonsson T (2013). The neural correlates of self-paced finger tapping in bipolar depression with motor retardation. Acta Neuropsychiatr.

[5] Genzel L, Dresler M, Cornu M (2015). Medial prefrontal-hippocampal connectivity and motor memory consolidation in depression and schizophrenia. Biol Psychiatry.

[6] Northoff G, Hirjak D, Wolf RC, Magioncalda P, Martino M (2021). All roads lead to the motor cortex: psychomotor mechanisms and their biochemical modulation in psychiatric disorders. Mol Psychiatry.

[7] Sarkheil P, Odysseos P, Bee I, Zvyagintsev M, Neuner I, Mathiak K (2020). Functional connectivity of supplementary motor area during finger-tapping in major depression. Compr Psychiatry.

[8] Roland PE, Larsen B, Lassen NA, Skinhoj E (1980). Supplementary motor area and other cortical areas in organization of voluntary movements in man. J Neurophysiol.

[9] Boschert J, Hink RF, Deecke L (1983). Finger movement versus toe movement-related potentials: further evidence for supplementary motor area (SMA) participation prior to voluntary action. Exp Brain Res.

[10] Jo HG, Habel U, Schmidt S (2019). Role of the supplementary motor area in auditory sensory attenuation. Brain Struct Funct.

[11] Nachev P, Kennard C, Husain M (2008). Functional role of the supplementary and pre-supplementary motor areas. Nat Rev Neurosci.

[12] Andrews-Hanna JR, Reidler JS, Sepulcre J, Poulin R, Buckner RL (2010). Functional-anatomic fractionation of the brain’s default network. Neuron.

[13] Rolls ET (2019). The cingulate cortex and limbic systems for emotion, action, and memory. Brain Struct Funct.

[14] Wu CW, Lin SHN, Hsu LM (2020). Synchrony between default-mode and sensorimotor networks facilitates motor function in stroke rehabilitation: a pilot fMRI study. Front Neurosci.

[15] Berman MG, Peltier S, Nee DE, Kross E, Deldin PJ, Jonides J (2011). Depression, rumination and the default network. Soc Cogn Affect Neurosci.

[16] Ho TC, Connolly CG, Henje Blom E (2015). Emotion-dependent functional connectivity of the default mode network in adolescent depression. Biol Psychiatry.

[17] Preuss A, Bolliger B, Schicho W (2020). SSRI treatment response prediction in depression based on brain activation by emotional stimuli. Front Psychiatry.

[18] Zhou HX, Chen X, Shen YQ (2020). Rumination and the default mode network: Meta-analysis of brain imaging studies and implications for depression. Neuroimage.

[19] World Health Organization (1993) The ICD-10 Classification of Mental and Behavioural Disorders: Diagnostic Criteria for Research. World Health Organization

[20] American Psychiatric Association (2013) American Psychiatric Association, eds. Diagnostic and Statistical Manual of Mental Disorders: DSM-5. 5th ed. American Psychiatric Association

[21] Ulbricht CM, Dumenci L, Rothschild AJ, Lapane KL (2018). Changes in depression subtypes among men in STAR*D: a latent transition analysis. Am J Mens Health.

[22] Janzing JGE, Birkenhäger TK, van den Broek WW, Breteler LMT, Nolen WA, Verkes RJ (2020). Psychomotor retardation and the prognosis of antidepressant treatment in patients with unipolar psychotic depression. J Psychiatr Res.

[23] Young KS, Parsons CE, Stein A, Kringelbach ML (2015). Motion and emotion: depression reduces psychomotor performance and alters affective movements in caregiving interactions. Front Behav Neurosci.

[24] Almeida JRC, Phillips ML (2013). Distinguishing between unipolar depression and bipolar depression: current and future clinical and neuroimaging perspectives. Biol Psychiatry.

[25] Downar J, Daskalakis ZJ (2013). New targets for rTMS in depression: a review of convergent evidence. Brain Stimulat.

[26] Swann AC, Katz MM, Bowden CL, Berman NG, Stokes PE (1999). Psychomotor performance and monoamine function in bipolar and unipolar affective disorders. Biol Psychiatry.

[27] van der Werf-Eldering MJ, Burger H, Holthausen EAE, Aleman A, Nolen WA (2010). Cognitive functioning in patients with bipolar disorder: association with depressive symptoms and alcohol use. PLoS ONE.

[28] Sasayama D, Hori H, Teraishi T (2012). More severe impairment of manual dexterity in bipolar disorder compared to unipolar major depression. J Affect Disord.

[29] Snyder HR (2013). Major depressive disorder is associated with broad impairments on neuropsychological measures of executive function: a meta-analysis and review. Psychol Bull.

[30] Blackburn IM (1975). Mental and psychomotor speed in depression and mania. Br J Psychiatry.

[31] Popescu C, Ionescu R, Jipescu I, Popa S (1991). Psychomotor functioning in unipolar and bipolar affective disorders. Romanian J Neurol Psychiatry Rev Roum Neurol Psychiatr.

[32] Walker MP, Brakefield T, Morgan A, Hobson JA, Stickgold R (2002). Practice with sleep makes perfect: sleep-dependent motor skill learning. Neuron.

[33] Walker MP, Brakefield T, Seidman J, Morgan A, Hobson JA, Stickgold R (2003). Sleep and the time course of motor skill learning. Learn Mem.

[34] Coffman JA, Bornstein RA, Olson SC, Schwarzkopf SB, Nasrallah HA (1990). Cognitive impairment and cerebral structure by MRI in bipolar disorder. Biol Psychiatry.

[35] Shinn AK, Yuksel C, Masters G (2019). Procedural memory consolidation after a night of sleep in bipolar disorder with psychotic features. Schizophr Res.

[36] Dresler M, Kluge M, Genzel L, Schüssler P, Steiger A (2010). Impaired off-line memory consolidation in depression. Eur Neuropsychopharmacol.

[37] Dresler M, Kluge M, Pawlowski M, Schüssler P, Steiger A, Genzel L (2011). A double dissociation of memory impairments in major depression. J Psychiatr Res.

[38] Genzel L, Ali E, Dresler M, Steiger A, Tesfaye M (2011). Sleep-dependent memory consolidation of a new task is inhibited in psychiatric patients. J Psychiatr Res.

[39] Hueng TT, Lee IH, Guog YJ (2011). Is a patient-administered depression rating scale valid for detecting cognitive deficits in patients with major depressive disorder?. Psychiatry Clin Neurosci.

[40] Lee CJ, Lee LT, Tsai HC (2018). Factors related to metabolic parameters in medicated patients with major depressive disorder––a naturalistic study. Psychiatry Res.

[41] Cantisani A, Stegmayer K, Bracht T (2016). Distinct resting-state perfusion patterns underlie psychomotor retardation in unipolar vs. bipolar depression. Acta Psychiatr Scand.

[42] Rock PL, Roiser JP, Riedel WJ, Blackwell AD (2014). Cognitive impairment in depression: a systematic review and meta-analysis. Psychol Med.

[43] Roca M, Monzón S, Vives M (2015). Cognitive function after clinical remission in patients with melancholic and non-melancholic depression: a 6 month follow-up study. J Affect Disord.

[44] Herszage J, Dayan E, Sharon H, Censor N (2020). Explaining individual differences in motor behavior by intrinsic functional connectivity and corticospinal excitability. Front Neurosci.

[45] Altinay MI, Hulvershorn LA, Karne H, Beall EB, Anand A (2016). Differential resting-state functional connectivity of striatal subregions in bipolar depression and hypomania. Brain Connect.

[46] Martino M, Magioncalda P, Conio B (2020). Abnormal functional relationship of sensorimotor network with neurotransmitter-related nuclei via subcortical-cortical loops in manic and depressive phases of bipolar disorder. Schizophr Bull.

[47] Magioncalda P, Martino M, Conio B (2020). Intrinsic brain activity of subcortical-cortical sensorimotor system and psychomotor alterations in schizophrenia and bipolar disorder: a preliminary study. Schizophr Res.

[48] Han S, He Z, Duan X (2019). Dysfunctional connectivity between raphe nucleus and subcortical regions presented opposite differences in bipolar disorder and major depressive disorder. Prog Neuropsychopharmacol Biol Psychiatry.

[49] Anand A, Jones SE, Lowe M, Karne H, Koirala P (2018). Resting state functional connectivity of dorsal raphe nucleus and ventral tegmental area in medication-free young adults with major depression. Front Psychiatry.

[50] Martino M, Magioncalda P (2021). Tracing the psychopathology of bipolar disorder to the functional architecture of intrinsic brain activity and its neurotransmitter modulation: a three-dimensional model. Mol Psychiatry.

[51] Conio B, Martino M, Magioncalda P (2020). Opposite effects of dopamine and serotonin on resting-state networks: review and implications for psychiatric disorders. Mol Psychiatry.

[52] Magioncalda P, Martino M (2021). A unified model of the pathophysiology of bipolar disorder. Mol Psychiatry.

[53] Martino M, Magioncalda P, Huang Z (2016). Contrasting variability patterns in the default mode and sensorimotor networks balance in bipolar depression and mania. Proc Natl Acad Sci.

[54] Russo D, Martino M, Magioncalda P, Inglese M, Amore M, Northoff G (2020). Opposing changes in the functional architecture of large-scale networks in bipolar mania and depression. Schizophr Bull.

[55] Oldfield RC (1971). The assessment and analysis of handedness: the Edinburgh inventory. Neuropsychologia.

[56] Uher R, Farmer A, Maier W (2008). Measuring depression: comparison and integration of three scales in the GENDEP study. Psychol Med.

[57] Milak MS, Keilp J, Parsey RV, Oquendo MA, Malone KM, Mann JJ (2010). Regional brain metabolic correlates of self-reported depression severity contrasted with clinician ratings. J Affect Disord.

[58] Phillips ML, Travis MJ, Fagiolini A, Kupfer DJ (2008). Medication effects in neuroimaging studies of bipolar disorder. Am J Psychiatry.

[59] Hassel S, Almeida JR, Kerr N (2008). Elevated striatal and decreased dorsolateral prefrontal cortical activity in response to emotional stimuli in euthymic bipolar disorder: no associations with psychotropic medication load. Bipolar Disord.

[60] Versace A, Almeida JRC, Hassel S (2008). Elevated left and reduced right orbitomedial prefrontal fractional anisotropy in adults with bipolar disorder revealed by tract-based spatial statistics. Arch Gen Psychiatry.

[61] Almeida JRC, Akkal D, Hassel S (2009). Reduced gray matter volume in ventral prefrontal cortex but not amygdala in bipolar disorder: significant effects of gender and trait anxiety. Psychiatry Res.

[62] Hassel S, Almeida JR, Frank E (2009). Prefrontal cortical and striatal activity to happy and fear faces in bipolar disorder is associated with comorbid substance abuse and eating disorder. J Affect Disord.

[63] Manoach DS, Thakkar KN, Stroynowski E (2010). Reduced overnight consolidation of procedural learning in chronic medicated schizophrenia is related to specific sleep stages. J Psychiatr Res.

[64] Pruim RHR, Mennes M, van Rooij D, Llera A, Buitelaar JK, Beckmann CF (2015). ICA-AROMA: a robust ICA-based strategy for removing motion artifacts from fMRI data. Neuroimage.

[65] Van Dijk KRA, Sabuncu MR, Buckner RL (2012). The influence of head motion on intrinsic functional connectivity MRI. Neuroimage.

[66] Hardwick RM, Rottschy C, Miall RC, Eickhoff SB (2013). A quantitative meta-analysis and review of motor learning in the human brain. Neuroimage.

[67] Beckmann CF, Smith SM (2004). Probabilistic independent component analysis for functional magnetic resonance imaging. IEEE Trans Med Imaging.

[68] Shaw ED, Stokes PE, Mann JJ, Manevitz AZ (1987). Effects of lithium carbonate on the memory and motor speed of bipolar outpatients. J Abnorm Psychol.

[69] Shimoyama I, Ninchoji T, Uemura K (1990). the finger-tapping test: a quantitative analysis. Arch Neurol.

[70] Calugi S, Cassano GB, Litta A (2011). Does psychomotor retardation define a clinically relevant phenotype of unipolar depression?. J Affect Disord.

[71] Jiménez-Jiménez FJ, Calleja M, Alonso-Navarro H (2011). Influence of age and gender in motor performance in healthy subjects. J Neurol Sci.

[72] Prigatano GP, Goncalves CWP, de Oliveira SB, Denucci SM, Pereira RM, Braga LW (2020). Kinematic recordings while performing a modified version of the Halstead finger tapping test: age, sex, and education effects. J Clin Exp Neuropsychol.

[73] Tzourio-Mazoyer N, Landeau B, Papathanassiou D (2002). Automated anatomical labeling of activations in SPM using a macroscopic anatomical Parcellation of the MNI MRI single-subject brain. Neuroimage.

[74] Arber S (2012). Motor circuits in action: specification, connectivity, and function. Neuron.

[75] Fischer S, Hallschmid M, Elsner AL, Born J (2002). Sleep forms memory for finger skills. Proc Natl Acad Sci.

[76] Caligiuri MP, Gentili V, Eberson S, Kelsoe J, Rapaport M, Gillin JC (2003). A quantitative neuromotor predictor of antidepressant non-response in patients with major depression. J Affect Disord.

[77] Pruim RHR, Mennes M, Buitelaar JK, Beckmann CF (2015). Evaluation of ICA-AROMA and alternative strategies for motion artifact removal in resting state fMRI. Neuroimage.

[78] Hirschfeld RMA, Vornik LA, Lewis L (2003). Perceptions and impact of bipolar disorder: how far have we really come? results of the national depressive and manic-depressive association 2000 survey of individuals with bipolar disorder. J Clin Psychiatry.

[79] Bluhm R, Williamson P, Lanius R (2009). Resting state default-mode network connectivity in early depression using a seed region-of-interest analysis: Decreased connectivity with caudate nucleus. Psychiatry Clin Neurosci.

[80] Fox MD, Snyder AZ, Vincent JL, Corbetta M, Van Essen DC, Raichle ME (2005). The human brain is intrinsically organized into dynamic, anticorrelated functional networks. Proc Natl Acad Sci U S A.

[81] Kelly AMC, Uddin LQ, Biswal BB, Castellanos FX, Milham MP (2008). Competition between functional brain networks mediates behavioral variability. Neuroimage.

[82] Biswal B, Yetkin FZ, Haughton VM, Hyde JS (1995). Functional connectivity in the motor cortex of resting human brain using echo-planar mri. Magn Reson Med.

[83] Xiong J, Parsons LM, Gao JH, Fox PT (1999). Interregional connectivity to primary motor cortex revealed using MRI resting state images. Hum Brain Mapp.

[84] Marek S, Tervo-Clemmens B, Calabro FJ (2022). Reproducible brain-wide association studies require thousands of individuals. Nature.

[85] Ghaemi SN, Sachs GS, Chiou MA, Pandurangi AK, Goodwin FK (1999). Is bipolar disorder still underdiagnosed? Are antidepressants overutilized?. J Affect Disord.

