# Supplementary material for: Motor performance and functional connectivity between the posterior cingulate cortex and supplementary motor cortex in bipolar and unipolar depression
Source: Eur Arch Psychiatry Clin Neurosci. 2023 Aug 28;274(3):655–71. doi: 10.1007/s00406-023-01671-1 (PMC10995093; doi:10.1007/s00406-023-01671-1)
Supplement: Supplementary file 1 — Supplementary file1 (DOCX 109 KB) [file 406_2023_1671_MOESM1_ESM.docx]

**Supplementary material for:**

**Motor performance and functional connectivity between the posterior cingulate cortex and supplementary motor cortex in bipolar and unipolar depression**

Lara E. Marten^a^, Aditya Singh (Dr. rer. nat)^a^, Anna M. Muellen^b^, Sören M. Noack^a^, Vladislav Kozyrev (Dr. rer. nat)^a,c,d^, Renate Schweizer (Dr. rer. nat)^c,e^, Roberto Goya-Maldonado (PD Dr. med.)^a,e^

**Author affiliations:**

^a^Laboratory of Systems Neuroscience and Imaging in Psychiatry (SNIP-Lab), Department of Psychiatry and Psychotherapy, University Medical Center Göttingen (UMG), Von-Siebold-Straße 5, 37075 Göttingen, Germany

^b^Cognitive Neuroscience Laboratory, German Primate Center, Kellnerweg 4, 37077 Göttingen, Germany

^c^Functional Imaging Laboratory, German Primate Center, Kellnerweg 4, 37077 Göttingen, Germany

^d^Institute of Molecular and Clinical Ophthalmology Basel, Mittlere Straße 91, 4056 Basel, Switzerland

^e^Leibniz ScienceCampus Primate Cognition, Kellnerweg 4, 37077 Göttingen, Germany

**Short running title:** PCC-SMA dysconnectivity in BD not UD

**Correspondence to:**

PD Dr. med. Roberto Goya-Maldonado

Universitätsmedizin Göttingen (UMG)

Klinik für Psychiatrie und Psychotherapie

Labor für systemische Neurowissenschaften und Bildgebung in der Psychiatrie (SNIP-Lab)

Von-Siebold-Str. 5

D-37075 Göttingen

Germany

Phone: +49 551-39-22244

E-mail: [roberto.goya@med.uni-goettingen.de](mailto:roberto.goya@med.uni-goettingen.de)

**Table of contents**

1. [Materials and methods 3](#_TOC_250008)
   1. [Medication load 3](#_TOC_250007)
2. [Results 7](#_TOC_250006)
   1. [Medication 7](#_TOC_250005)
   2. [Quality check new seed definition technique 8](#_TOC_250004)

[2.2.3 Right hand motor cortex (M1) seeds 8](#_TOC_250003)

[2.2.3 Posterior cingulate cortex (PCC) seeds 9](#_TOC_250002)

- 1. [Frame-by-frame displacements comparison 10](#_TOC_250001)
  2. Group and time comparisons SMN-DMN correlation coefficients 11

1. [References supplementary material 13](#_TOC_250000)

# Materials and methods

## Medication load

The measurement of medications was performed, whenever possible, in blood and as close as possible to the MRI measurement, but at least 10 hours apart from the last intake to account for first pass metabolization. The reference ranges are given by the central laboratory of the University Medical Center Göttingen (UMG) and complemented by the online version of the Rote Liste (May 2020), if necessary. The medication load was computed as a composite of the sum of ranges,1–5 varying from below (coded as 1), within (coded as 2) and above (coded as 3) the reference range (Table 1). Exceptions were hormones, excluding Melatonin, and lithium, if blood measurement as part of the study failed, as these are medications individually dosed by the caregivers according to blood levels (coded as 2). Not regularly taking medication or medication that was not considered to be psychoactive (see Table 2), the code was 0.

*Table 1: Medication with reference ranges for medication load*

| **Substance class** | **Substance** | **Metabolite in blood or**  **Rote Liste indication and administration** | **Reference range** | | |
| --- | --- | --- | --- | --- | --- |
|  |  |  | **Min.** | **Max.** | **Unit** |
| Anticholinergic | Biperiden | Pills and retard pills | 3 | 16 | mg |
| Antidepressant | Agomelatine | Pills | 25 | 50 | mg |
|  | Amitriptyline | Blood level Amitriptyline  + Nortriptyline | 80 | 200 | µg/L |
|  | Amitriptyline | Pills and retard pills | 50 | 150 | mg |
|  | Bupropion | Pills and retard pills | 150 | 300 | mg |
|  | Citalopram | Blood level Citalopram | 50 | 110 | µg/L |
|  | Citalopram | Treatment of depression, pills | 20 | 40 | mg |
|  | Duloxetine | Blood level Duloxetine | 30 | 120 | µg/L |
|  | Duloxetine | Treatment of depression, enteric-coated hard capsule | 60 | 60 | mg |
|  | Escitalopram | Blood level Escitalopram | 15 | 80 | µg/L |
|  | Escitalopram | Treatment of major depressive disorder, pills | 10 | 20 | mg |
|  | Mirtazapine | Blood level Mirtazapine | 30 | 80 | µg/L |
|  | Mirtazapine | Pills | 15 | 45 | mg |
|  | Sertraline | Blood level Sertraline | 10 | 150 | µg/L |
|  | Sertraline | Treatment of depression and obsessive-compulsive disorder, pills | 50 | 250 | mg |
|  | Tianeptine | Pills | 37,5 | 37,5 | mg |
|  | Trazodone | Outpatient treatment, pills | 200 | 400 | mg |

| **Substance class** | **Substance** | **Metabolite in blood or Rote Liste indication and administration** | **Reference range** | | |
| --- | --- | --- | --- | --- | --- |
|  |  |  | **Min.** | **Max.** | **Unit** |
|  | Venlafaxine | Blood level Venlafaxine + O-Desmethylvenlafaxine | 100 | 400 | µg/L |
|  | Venlafaxine | Treatment of major  depressive disorder, pills or retard pills | 75 | 375 | mg |
| Antihistaminic | Promethazine | Long term treatment, pills | 20 | 100 | mg |
| Anticonvulsive | Lamotrigine | Blood level Lamotrigine | 2 | 10 | mg/L |
|  | Lamotrigine | Optimal dose bipolar disorder, pills | 200 | 200 | mg |
|  | Pregabalin | Blood level Pregabalin | 2 | 5 | mg/L |
|  | Pregabalin | Treatment of generalized anxiety disorder, hard capsule | 150 | 600 | mg |
| Benzodiazepine | Diazepam | Outpatient treatment, pills | 5 | 10 | mg |
|  | Lorazepam | Sleep disorders due to anxiety and inner tension, pills | 0,5 | 2,5 | mg |
| Dopamin agonists | Rotigotine | Restless-Legs-syndrome transdermal patch, dose per 24h | 1 | 3 | mg |
| Hormones | Dehydro-  epiandrosteron | Substitution of hormones | individual | | |
|  | Hydrocortison e | Substitution of hormones | individual | | |
|  | L-Thyroxin | Substitution of hormones | individual | | |
|  | Melatonin | Retard pills | 2 | 2 | mg |
| Mood stabilizer | Lithium | Blood level Lithium | 0,6 | 1,2 | mmol/L |
|  | Lithium | Pills | Reference after blood level only | | |
|  | Valproate acid | Blood level Valproate acid (amedes MVZ wagnerstibbe für Laboratoriumsmedizin) | 50 | 100 | mg/L |
|  | Valproate acid | Blood level Valproate acid (central laboratory UMG) | 346 | 696 | µmol/L |
|  | Valproate acid | Bipolar disorder, different  galenic | 1000 | 2000 | mg |
| Muscle relaxer | Baclofen | Pills | 30 | 75 | mg |
| Neuroleptic | Aripiprazole | Blood level Aripiprazole | 150 | 500 | µg/L |

| **Substance class** | **Substance** | **Metabolite in blood or Rote Liste indication and administration** | **Reference range** | | |
| --- | --- | --- | --- | --- | --- |
|  |  |  | **Min.** | **Max.** | **Unit** |
|  | Aripiprazole | Manic episodes of Bipolar- I-Disorder, pills | 15 | 30 | mg |
|  | Benperidol | Maintenance dose, pills | 1 | 6 | mg |
|  | Haloperidol | Blood level Haloperidol | 1 | 10 | µg/L |
|  | Haloperidol | Treatment of moderate to severe manic episodes in  bipolar-I-disorder, drops | 2 | 10 | mg |
|  | Olanzapine | Blood level Olanzapine | 20 | 80 | µg/L |
|  | Olanzapine | Treatment of bipolar disorder, pills | 5 | 20 | mg |
|  | Pipamperone | Minimum for sleep disorder, Maximum general use, pills | 40 | 360 | mg |
|  | Quetiapine | Blood level Quetiapine | 100 | 500 | µg/L |
|  | Quetiapine | Ad-on-treatment for major depressive disorder, retard pills | 50 | 300 | mg |
|  | Quetiapine | Treatment of severe depressive episodes in bipolar disorder, retard pills | 300 | 300 | mg |
|  | Quetiapine | Treatment of severe depressive episodes, pills | 300 | 300 | mg |
|  | Risperidone | Blood level Risperidone +  9-OH-Risperidone | 20 | 60 | µg/L |
|  | Risperidone | Treatment of mania in bipolar disorder, pills | 1 | 6 | mg |
| Non- Benzodiazepines | Zolpidem | Pills | 10 | 10 | mg |
|  | Zopiclone | Pills | 7,5 | 7,5 | mg |
| Opioid agonists | Oxycodone/ Naloxone | Retard pills | 20/  10 | 20/  10 | mg |

The reference ranges above are provided by the central laboratory of the UMG and complemented by the digital version of Rote Liste (https:/[/www](http://www.rote-liste.de/)).[rote-liste.de/),](http://www.rote-liste.de/)) Liste Arzneimittelinformation für Deutschland).

*Table 2: Medication not considered as psychotropic*

| Acetylsalicylic |
| --- |
| Allopurinol |
| Amlodipine |
| Bisoprolol |
| Candesartan |
| Cholecalciferol |
| Diclofenac |
| Dihydralazine |
| Disulfiram |
| Doxazosin |
| Enalapril |
| Etoricoxib |
| Fluvastatin |
| Folic acid |
| Hydrochlorothiazide |
| Ibuprofen |
| Kalium |
| Lercanidipine |
| Metoprolol |
| Novaminsulfon |
| Omeprazole |
| Pantoprazole |
| Phenprocoumon |
| Ramipril |
| Spironolactone |
| Torsemide |
| Valsartan |
| Mixture of B-Vitamins |
| Vitamin B1 |

# Results

## Medication

*Table 3: Number of unipolar (UD) and bipolar (BD) depression patients per antidepressant in sessions 1 and 2*

| **Substance** | **UD1** | **BD1** | **UD2** | **BD2** |
| --- | --- | --- | --- | --- |
| Agomelatine | 1 | 0 | 2 | 0 |
| Amitriptyline | 2 | 0 | 3 | 0 |
| Bupropion | 4 | 0 | 4 | 0 |
| Citalopram | 0 | 1 | 0 | 0 |
| Duloxetine | 5 | 2 | 4 | 2 |
| Escitalopram | 4 | 0 | 3 | 0 |
| Mirtazapine | 7 | 2 | 6 | 1 |
| Sertraline | 9 | 2 | 6 | 2 |
| Tianeptine | 1 | 0 | 1 | 0 |
| Trazodone | 1 | 0 | 1 | 0 |
| Venlafaxine | 4 | 7 | 7 | 6 |

The number of patients taking at least one neuroleptic was comparable between the groups (visit 1: UD = 12, BD = 13; visit 2: UD = 12, BD = 12).

*Table 4: Number of unipolar (UD) and bipolar (BD) depression patients per neuroleptic in sessions 1 and 2*

| **Substance** | **UD1** | **BD1** | **UD2** | **BD2** |
| --- | --- | --- | --- | --- |
| Aripiprazole | 0 | 1 | 0 | 1 |
| Benperidol | 0 | 1 | 0 | 0 |
| Haloperidol | 0 | 0 | 0 | 1 |
| Olanzapine | 0 | 2 | 0 | 1 |
| Pipamperone | 0 | 0 | 1 | 0 |
| Quetiapine | 9 | 8 | 8 | 9 |
| Risperidone | 3 | 3 | 4 | 1 |

*Table 5: Number of unipolar (UD) and bipolar (BD) depression patients per other psychotropic medication in sessions 1 and 2*

| **Substance class** | **Substance** | **UD1** | **BD1** | **UD2** | **BD2** |
| --- | --- | --- | --- | --- | --- |
| Anticholinergic | Biperiden | 0 | 1 | 0 | 1 |
| Antihistaminic | Promethazine | 0 | 2 | 0 | 2 |
| Anticonvulsive | Lamotrigine | 1 | 2 | 2 | 1 |
|  | Pregabalin | 2 | 1 | 2 | 0 |
| Benzodiazepine | Diazepam | 1 | 2 | 1 | 1 |
|  | Lorazepam | 5 | 2 | 4 | 2 |
| Dopamine agonists | Rotigotine | 1 | 0 | 1 | 0 |
| Hormones | Dehydroepi- androsteron | 1 | 0 | 1 | 0 |
|  | Hydrocortisone | 1 | 0 | 1 | 0 |
|  | L-Thyroxin | 2 | 2 | 2 | 2 |
|  | Melatonin | 1 | 1 | 0 | 1 |
| Mood stabilizer | Lithium | 2 | 9 | 2 | 11 |
|  | Valproate acid | 0 | 3 | 0 | 4 |
| Muscle relaxer | Baclofen | 0 | 1 | 0 | 0 |
| Non- Benzodiazepines | Zolpidem | 0 | 1 | 0 | 0 |
|  | Zopiclone | 1 | 0 | 0 | 0 |
| Opioid/Opioid- antagonists | Oxycodone/ Naloxone | 1 | 0 | 1 | 0 |

## Quality check new seed definition technique

### 2.2.3 Right hand motor cortex (M1) seeds

No difference in the volume of individual M1 seeds was seen between groups (F(2,76) = 0.649; p = 0.525) or across time (F(1,76) = 0.218; p = 0.642) (Fig. 1, supplementary material). Surprisingly, there was a significant interaction between group and time (F(2,76) = 3.774; p = 0.027), but, since a comparison of the extend of functional changes across the groups was out of the scope of this work, this interaction is out of relevance for potential bias. These results remained the same when excluding the lefthanders from analysis.


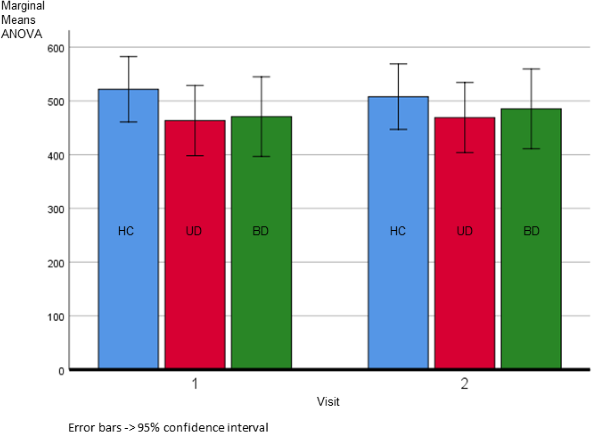


*Figure 1. Marginal means and confidence intervals from repeated-measures ANOVA of right hand motor cortex seed size (in mm3) for healthy controls (HC, blue), unipolar (UD, red) and bipolar (BD, green) patients. HC visit 1 M ± SD = 521.8 ± 139.6 mm3, UD visit 1 M ± SD = 463.4 ± 185.7 mm3, BD visit 1 M ± SD = 470.9 ± 189.6 mm3, HC visit 2 M ± SD = 507.9 ±*

*128.6 mm3, UD visit 2 M ± SD = 469.0 ± 189.4 mm3, BD visit 2 M ± SD = 485.3 ± 197.5 mm3*

### 2.2.3 Posterior cingulate cortex (PCC) seeds

No difference in the volume of individual PCC seeds was seen between groups (F(2,76) = 1.130; p = 0.328) or across time (F(1,76) = 0.133; p = 0.716) (Fig. 2 supplementary material). Also, there was no interaction seen between group and time (F(2,76) = 0.886; p = 0.417). These results remained the same when excluding the lefthanders from analysis.


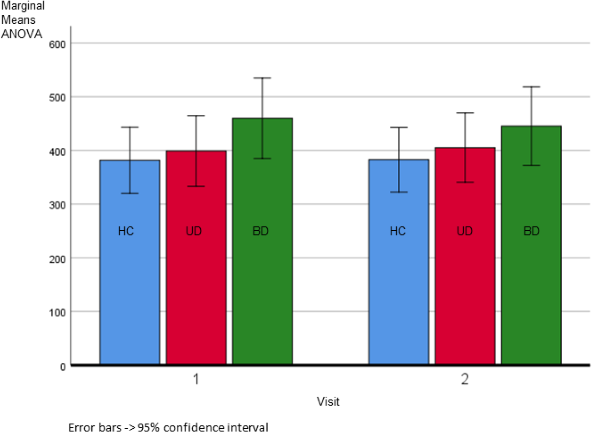


*Figure 2. Marginal means and confidence intervals from repeated-measures ANOVA of posterior cingulate cortex seed size (in mm3) for healthy controls (HC, blue), unipolar (UD, red) and bipolar (BD, green) patients. HC visit 1 M ± SD = 381.4 ± 146.8 mm3, UD visit 1 M*

*± SD = 398.8 ± 157.7 mm3, BD visit 1 M ± SD = 459.8 ± 219.0 mm3, HC visit 2 M ± SD = 382.5 ± 140.1 mm3, UC visit 2 M ± SD = 405.3 ± 156.0 mm3, BD visit 2 M ± SD = 445.3 ±*

*215.3 mm3*

## Frame-by-frame displacements comparison

In the rmANOVA of the individual root-mean-squares of the frame-by-frame displacement a difference between groups was seen (F(2,76) = 3.716; p = 0.029) (Fig. 3, supplementary material), although the Games-Howell-post-hoc tests (applied because assumption of homogeneity of variance was not met) did not show a significant difference (HC-UD p = 0.519; HC-BD p = 0.257; UD-BD p = 0.062). There was neither a significant effect of time (F(1,76) = 0.002; p = 0.969) seen nor a group x time interaction (F(2,76) = 0.297; p = 0.744).


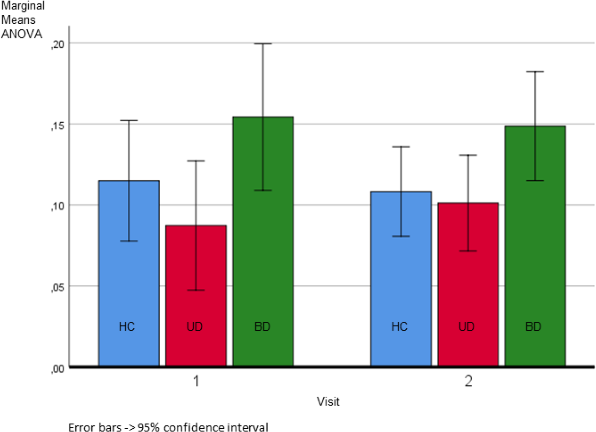


*Figure 3. Marginal means and confidence intervals from repeated-measures ANOVA of root- mean-squares of the frame-by-frame displacement (in mm) for healthy controls (HC, blue), unipolar (UD, red) and bipolar (BD, green) patients. HC visit 1 M ± SD = 0.12 ± 0.08 mm, UD visit 1 M ± SD = 0.09 ± 0.06 mm, BD visit 1 M ± SD = 0.15 ± 0.16 mm, HC visit 2 M ± SD = 0.11 ± 0.07 mm, UC visit 2 M ± SD = 0.10 ± 0.08 mm, BD visit 2 M ± SD = 0.15 ± 0.09 mm*

- 1. Group and time comparisons sensory motor network (SMN) - default mode network (DMN) correlation coefficients

*Table 6: Group and time comparison of SMN-DMN correlation coefficients*

| **HC visit 1 M ± SD** | **UD visit 1 M ± SD** | **BD visit 1 M ± SD** | **HC visit 2 M ± SD** | **UD visit 2 M ± SD** | **BD visit 2 M ± SD** | **rmANOVA factors** |
| --- | --- | --- | --- | --- | --- | --- |
| **posterior DMN-SMN (left handers included)** | | | | | | |
| -0.23 ± | -0.09 ± | -0.10 ± | -0.12 ± | -0.16 ± | 0.01 ± | Group: |
| 0.24 | 0.25 | 0.26 | 0.23 | 0.19 | 0.21 | F(2,76) = 3.031, p = |
|  |  |  |  |  |  | 0.054, |
|  |  |  |  |  |  | group effect driven by |
|  |  |  |  |  |  | BD-HC (Post-Hoc p = |
|  |  |  |  |  |  | 0.049) |

|  |  |  |  |  |  | Time:  F(1,76) = 2.328, p = 0.131  Group x Time: F(2,76) = 3.232, p = 0.045 |
| --- | --- | --- | --- | --- | --- | --- |
| **anterior DMN-SMN (left handers included)** | | | | | | |
| -0.01 ± | -0.13 ± | -0.16 ± | -0.08 ± | = -0.18 ± | 0.01 ± | Group: |
| 0.23 | 0.21 | 0.21 | 0.18 | 0.18 | 0.14 | F(2,76) = 3.571, p = |
|  |  |  |  |  |  | 0.033, |
|  |  |  |  |  |  | group effect driven by |
|  |  |  |  |  |  | UD-HC (Post-Hoc p = |
|  |  |  |  |  |  | 0.032) |
|  |  |  |  |  |  | Time: |
|  |  |  |  |  |  | F(1,76) = 0.327, p = |
|  |  |  |  |  |  | 0.569 |
|  |  |  |  |  |  | Group x Time: |
|  |  |  |  |  |  | F(2,76) = 6.760, p = |
|  |  |  |  |  |  | 0.002 |
| **DMN-SMN (left handers excluded, gICAs did not show a split of DMN)** | | | | | | |
| -0.16 ± | -0.22 ± | -0.33 ± | -0.10 ± | -0.24 ± | -0.10 ± | Group: |
| 0.23 | 0.27 | 0.16 | 0.21 | 0.21 | 0.20 | F(2,72) = 2.603, |
|  |  |  |  |  |  | p = 0.081 |
|  |  |  |  |  |  | Time: |
|  |  |  |  |  |  | F(1,72) = 8.710, |
|  |  |  |  |  |  | p = 0.004 |
|  |  |  |  |  |  | Group x Time: |
|  |  |  |  |  |  | F(2,72) = 4.588, |
|  |  |  |  |  |  | p = 0.013 |

*HC = Healthy controls, UD = unipolar group, BD = bipolar group, rmANOVA = repeated measures ANOVA*

# References

1. Phillips ML, Travis MJ, Fagiolini A, Kupfer DJ. Medication Effects in Neuroimaging Studies of Bipolar Disorder. *Am J Psychiatry*. 2008;165(3):313-320. doi:10.1176/appi.ajp.2007.07071066
2. Hassel S, Almeida JR, Kerr N, et al. Elevated striatal and decreased dorsolateral prefrontal cortical activity in response to emotional stimuli in euthymic bipolar disorder: no associations with psychotropic medication load. *Bipolar Disord*. 2008;10(8):916-927. doi:10.1111/j.1399-5618.2008.00641.x
3. Versace A, Almeida JRC, Hassel S, et al. Elevated Left and Reduced Right Orbitomedial Prefrontal Fractional Anisotropy in Adults With Bipolar Disorder Revealed by Tract-Based Spatial Statistics. *Arch Gen Psychiatry*. 2008;65(9):1041-1052. doi:10.1001/archpsyc.65.9.1041
4. Almeida JRC, Akkal D, Hassel S, et al. Reduced gray matter volume in ventral prefrontal cortex but not amygdala in bipolar disorder: significant effects of gender and trait anxiety. *Psychiatry Res*. 2009;171(1):54-68. doi:10.1016/j.pscychresns.2008.02.001
5. Hassel S, Almeida JR, Frank E, et al. Prefrontal cortical and striatal activity to happy and fear faces in bipolar disorder is associated with comorbid substance abuse and eating disorder. *J Affect Disord*. 2009;118(1-3):19-27. doi:10.1016/j.jad.2009.01.021
